# Agroclimatic landscapes of pergamon: Modeling agricultural suitability of an ancient city and its environs

**DOI:** 10.1371/journal.pone.0325779

**Published:** 2025-07-02

**Authors:** Robert Busch, Anne Dallmeyer, Ulrike Herzschuh, Felix Pirson, Brigitta Schütt, Fabian Becker

**Affiliations:** 1 Department of Earth Sciences, Institute of Geographical Sciences, Freie Universität Berlin, Berlin, Germany; 2 Max Planck Institute for Meteorology (MPI), Hamburg, Germany; 3 Alfred-Wegener-Institute for Polar and Marine Research (AWI), Potsdam, Germany; 4 Institute of Environmental Science and Geography, University of Potsdam, Potsdam, Germany; 5 Institute of Biochemistry and Biology, University of Potsdam, Potsdam, Germany; 6 Istanbul Department, Deutsches Archäologisches Institut (DAI), Istanbul, Türkiye; Israel Antiquities Authority, ISRAEL

## Abstract

The relationship between humans and the natural environment is shaped by the perception, utilization, and management of natural resources. In the Mediterranean region, the cultivation of resources has played a crucial role in shaping landscapes over time. Assessing the potential of landscapes for using various natural resources provides insights into the functioning of socio-ecological systems and highlights areas susceptible to environmental fluctuations and human exploitation. The environs of Pergamon – the ancient capital of the Pergamene Kingdom and later a major Roman city in Asia Minor – serve as an exemplary case of human-environment interaction in Western Anatolia. This work reconstructs the climatic potential for cultivating annual crops commonly used in Mediterranean rainfed agricultural systems, focusing on the Pergamon micro-region. Paleoclimate simulations from MPI-ESM 1.2 with high spatial resolution T63 (~1.875° x 1.875° on a gaussian grid) were integrated into the EcoCrop niche model to explore spatio-temporal changes in agricultural suitability from 400 BCE to 400 CE. The results highlight the consistent agricultural potential of the western Bakırçay plain due to its favorable climatic conditions. In contrast, the eastern plain and surrounding foothills exhibit greater variability, necessitating adaptive land management strategies. Comparisons with a pollen-based reconstruction confirmed the general alignment in climatic trends, supporting the plausibility of the modeled scenarios. Favorable conditions may have sustained agricultural productivity but also highlight dependencies on regional trade networks, decoupling Pergamon’s economy from local subsistence agriculture and introducing vulnerabilities to trade disruptions. This study challenges the notion of a uniform “Roman” Climate Optimum, highlighting the importance of understanding local agroclimatic conditions and adaptive land management strategies in ancient societies.

## 1 Introduction

The coasts of the Aegean Sea are historically rich landscapes where human civilizations have been and continue to be closely connected to complex ecosystem dynamics [1–3]. The perception, understanding, and subsequent use of natural resources in these ecosystems established fundamental connections, i.e., socio-ecological systems, between humans and the environment [4,5]. The establishment of socio-ecological systems involves a succession of adaptive and resilient processes driven by the mutual transformation of landscapes and societies [4,6,7], closely tied to the cultivation and management of resources [5]. Here, we understand the potential use of natural materials - such as soil, stone, and ore - as a cultivation process based on the perception and knowledge of how to utilize these materials and transform them into value.

With progressing settlement dynamics and population growth, the demand for natural resources such as arable land, water, and wood and, therefore, social metabolism [8,9] steadily increased. The resulting pressure on the landscape and its ability to resist changes (i.e., landscape sensitivity [10]) is described in several studies for the Aegean region and adjacent areas [11–14], highlighting an intensification of resource use and material flows within the socio-ecological system during the mid- to late Holocene. In addition to these pressures on the landscape, climatic changes have been a persistent force on the control and coupling of socio-ecological processes around the Aegean Sea [15,16]. For example, increased precipitation contributed to soil erosion and land degradation while enhancing opportunities for rain-fed agriculture. Ancient societies were closely linked to the dynamic environmental conditions of their surroundings, adopting land-use practices that reflected both short- and long-term changes in the landscape’s agricultural potential [2,17,18]. Climatic conditions, alongside terrain and soil quality, were among the most decisive challenges for the long-term planning of agriculture. However, it is necessary to consider a complex system of parameters that can influence plant growth in many different ways [15]. From ancient scholars such as Pliny the Elder, Cato the Elder, or Virgil, it is known that ancient societies had a good understanding of agricultural potentials and natural influences on cultivation [19–21].

**Fig 1:**
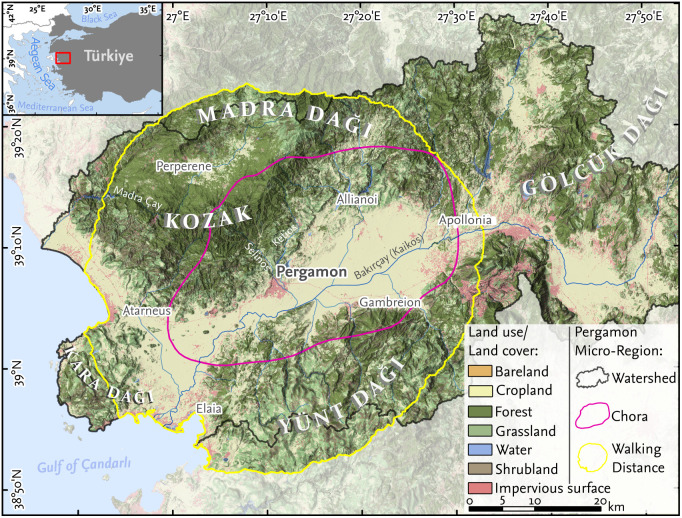
Overview of the Aegean coast and the location of Pergamon. The current land cover and land use [38] are shown with three different analytical spatial boundaries of the Pergamon micro-region: the watersheds of the Bakırçay and the Madra Çay [39], the *chora* of Pergamon during the Roman Imperial period [30], and the walking distance that can be reached within two days (16 **h)** [29]. Elevation data (hillshade) is based on TanDEM-X data [40]. Sites are labeled directly at their geographic locations.

**Fig 2:**
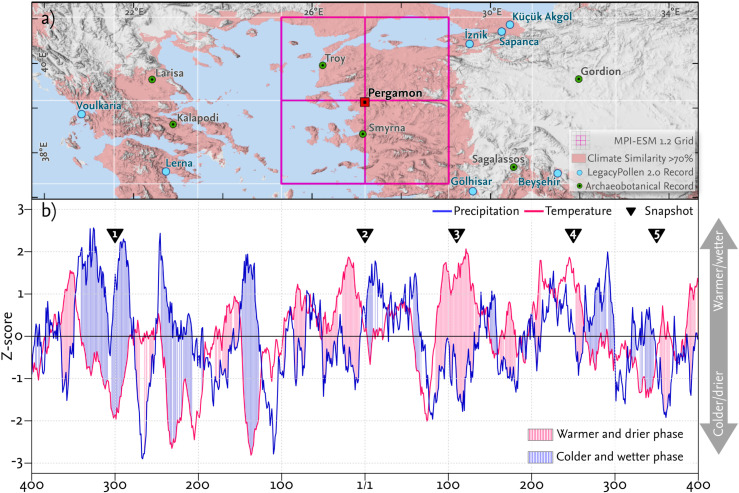
Regional setting and reconstructed climatic trends for western Anatolia and the Aegean. **a)** Overview of the data from the wider region around the Aegean Sea used in the current study. grid cells of the MPI-ESM 1.2 model; climatic similarity index for the present climatic situation in Bergama, based on WorldClim 2.1 data (see Section 3.3.2). Selected LegacyPollen 2.0 [57] and archaeobotanical records [58,59] are highlighted (see sections 3.2 and 3.3.4 for details). The records from Sagalassos were obtained from Düzen Tepe, and the records around Larisa were taken from Kompoloi, Krania, and Plantania. Elevation data (hillshade) is based on TanDEM-X data [40]. **b)** Climate variations are presented as z-scores for the period from 400 BCE to 400 CE for the northern Aegean region using a 20-year moving average of annual MPI-ESM 1.2 values. Phases of higher-than-average precipitation (z-score > 0) and lower-than-average temperature (z-scores <0) are classified as “colder and wetter”; phases of lower-than-average precipitation (z-score <0) and higher-than-average temperature (z-score >0) are classified as “warmer and drier”.

To explore the complex relationship between societies, natural resources, and climate around the Aegean Sea, this study examines past climatic changes in the region with its influence on the natural agricultural potential for cultivation in the environs of the ancient city of Pergamon (Asia Minor; today Aegean Region, Türkiye). According to the Climate Similarity Index for the city of Pergamon (today Bergama; Fig 1), almost the entire Aegean region shares today up to 70% of the same climatic variations (Fig 2a - see section 3.3.2), making the region an appropriate analog for studying climate impacts on archaeological sites throughout the Aegean. The chronological framework in this work is the period from the 4th century BCE to the 4th century CE, which encompasses the profound socio-ecological transformation of the Pergamon micro-region from the Hellenistic to the Roman Imperial period and Late Antiquity [22]. Despite increased research efforts, climate change during this period is still not fully understood across the Aegean and Mediterranean regions [23–26]. Theories such as the “Roman” Climate Optimum (RCO) [27], which is described as a phase of climatic stability and increased humidity, are subject to critical examination [28].

Previous research related to the agricultural potential of the Pergamon micro-region focused on its carrying capacity under different population and labor scenarios [29]. However, employing only static topographic parameters as the main limiting factor for agriculture, without accounting for temporal changes or climatic impacts on agricultural potential. Additionally, the Bakırçay plain, the receiving floodplain in the Pergamon micro-region, was described as fertile land in antiquity, featuring different land use patterns, possibly resulting from heterogeneous natural potentials [30].

In this work, we focus on modeling agricultural potential by assessing the cultivation suitability of different crops and legumes for rain-fed agriculture, incorporating climatic, topographic, and soil parameters, and applying the EcoCrop model [31,32]. EcoCrop was initially established by the Food and Agriculture Organization (FAO) as a database and tool for mitigating risks associated with climate change, enabling adaptation strategies that align with the natural capabilities of various crops [31]. The database and model are, therefore, widely applied in predicting scenarios under future climate change [33–35], but have not been integrated into hindcast climate change simulations to date. Although Goodchild et al. (2019) [36] aimed to reconstruct historical agricultural potentials in central Italy, their work relied on modern climate data, assuming similar past climatic conditions.

Our study incorporates climatic variables derived from hindcast climate simulations into the analysis of agricultural potential, introducing a temporal dimension by considering how changing temperature and precipitation patterns affect the suitability for cultivating specific crops over time. We, therefore, address the static limitations of previous agricultural potential models by utilizing a transient paleoclimate simulation from the Max Planck Institute Earth System Model version 1.2 (MPI-ESM 1.2) [37], assessing the evolving suitability of various crops in the Pergamon micro-region.

With this approach, our study investigates how climate fluctuations influenced the agroclimatic suitability for staple crops in the Pergamon region between 400 BCE and 400 CE, and how this regional pattern contributes to the ongoing discussion about the extent and character of the so-called “Roman” Climate Optimum.

## 2 Study area

Pergamon was one of the culturally most important Hellenistic and Roman cities around the Aegean Sea [41]. The remains of the ancient capital are located in the present-day city of Bergama [39° 8′ N, 27°11′ E], approximately 25 km inland from the Aegean coast, Türkiye (Izmir Province), in the far west of Anatolia (Fig 1). During Archaic times, Pergamon was carved and built on a promontory of the Kozak mountains and expanded during Hellenistic and Roman times on the adjacent alluvial fan of the Selinos (today Bergama River) and Ketios River (today Kestel River), as well as the slopes of adjacent hills [42].

The spatial delimitation of the study area around Pergamon is based on the overlay of multiple spatial definitions of the Pergamon micro-region (Fig 1) that is based on both physical (watersheds) and social domains (*chora*, walking territory), indicating presumable areas of socio-ecological interactions [22,29,30].

### 2.1 Natural settings

The Pergamon micro-region is characterized by a Mediterranean climate (Csa) with hot, dry summers and mild, wet winters (Köppen–Geiger classification [43]). The annual precipitation cycle exhibits an unimodal rainfall distribution, with its monthly minimum occurring in August (5 mm on average) and its maximum in December (103 mm on average). The rainy season lasts from October to March (Fig 2b). The annual precipitation sums up to 620 mm. The mean annual temperature is 16.7 °C (World Meteorological Organization Station 17742 in Bergama for the period of 1991–2020 [44]). Coastal areas around Dikili in the vicinity of the ancient Atarneus and Zeytindağ, close to ancient Elaia, experience maritime influences with higher average annual temperatures. In contrast, the hinterland around Paşaköy, located near ancient Allianoi, exhibits more continental temperature patterns (Fig 7). The regional wind patterns are mainly influenced by the Etesian winds (tur *meltem*); the strong, dry, northerly winds typically occur from mid-May to mid-September and play a dominant role in shaping the weather system around the Aegean Sea [45].

**Fig 3:**
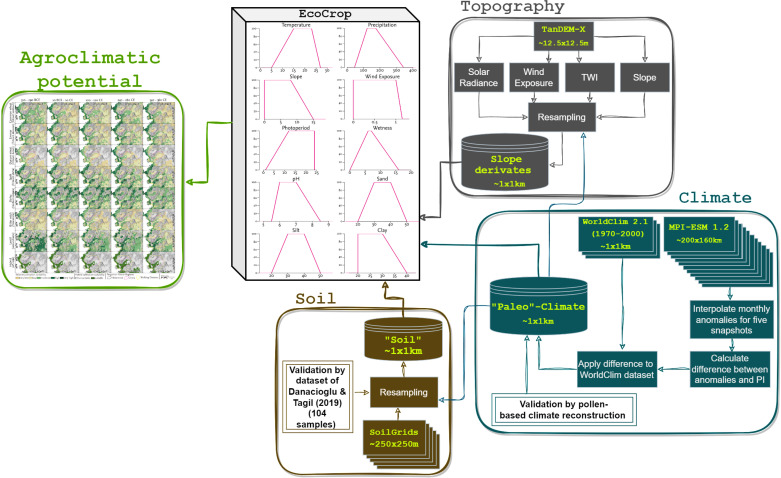
Overview of the agroclimatic potential modeling framework. The model incorporates climate, topography, and soil data at a resolution of approx. 1 x 1 km. The EcoCrop parameters comprised resampled soil, topographical, and climatic inputs derived from TanDEM-X, SoilGrids, WorldClim 2.1, and the MPI-ESM 1.2 (paleo-climate model). The soil data was validated using field data from [67]. Climate anomalies are interpolated over five snapshots, with the outputs validated by pollen-based climate reconstructions derived from LegacyPollen 2.0 records [57].

**Fig 4:**
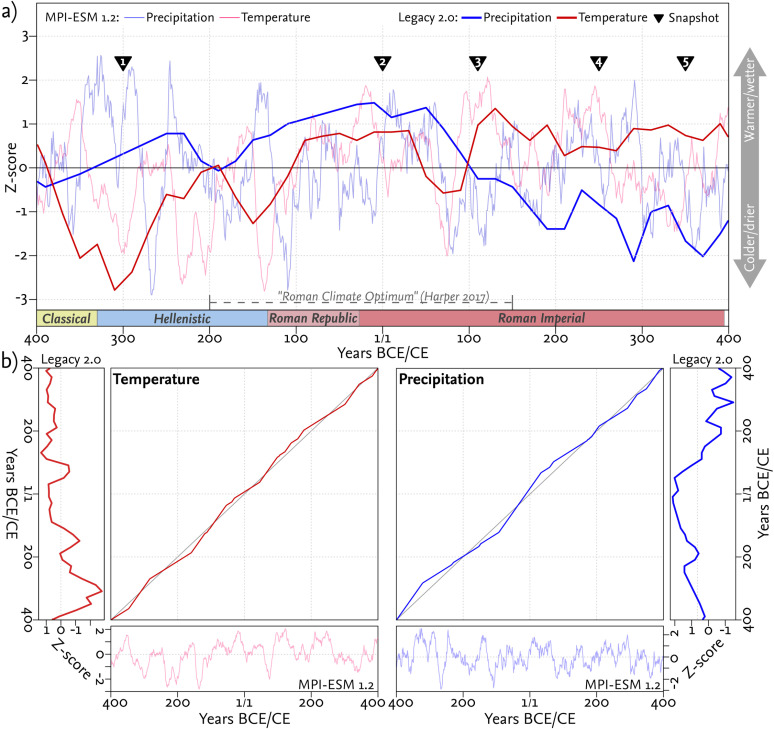
Comparison of pollen-based climate reconstructions and MPI-ESM 1.2 simulations. **a)** Comparison between z-scores of modeled climate variables from the MPI-ESM 1.2 simulation and the pollen-based climate reconstruction between 400 BCE and 400 CE. Both models are compared using the dynamic time warping (dtw) algorithm. **b)** Alignment paths between both reconstructions for temperature and precipitation values.

**Fig 5:**
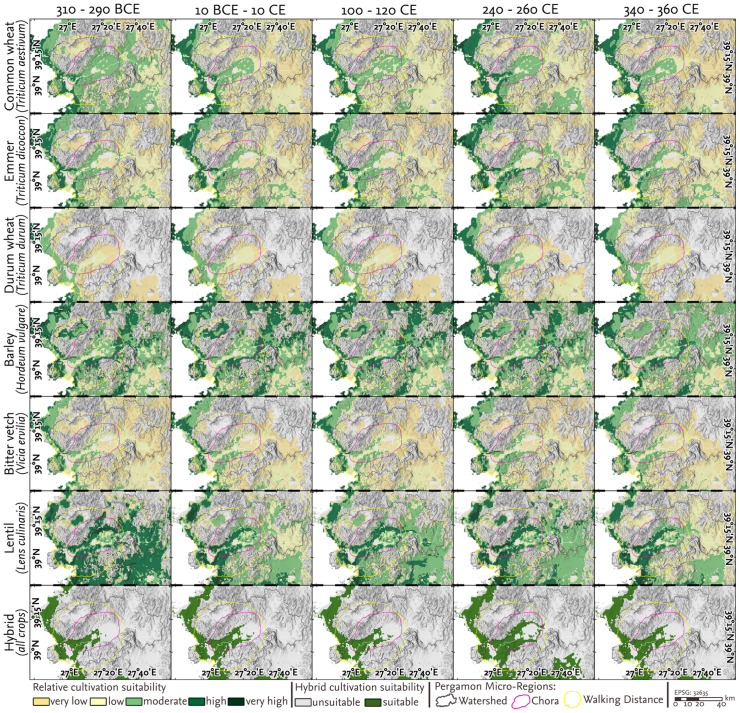
Overview of the six most conventional crops in the environs of Pergamon. Changes in cultivation suitability over the selected five time periods (310-290 BCE, 10 BCE-10 CE, 100-120 CE, 240-260 CE, 340-360 CE), highlighting periods with different climate conditions. The hybrid model incorporates the modeled suitability of the six conventional crop types, including four cereal and two legume types. The crop-specific outputs were trimmed to prevent overrepresenting nearly unsuitable areas in the hybrid model, considering only suitability estimations >20%.

**Fig 6:**
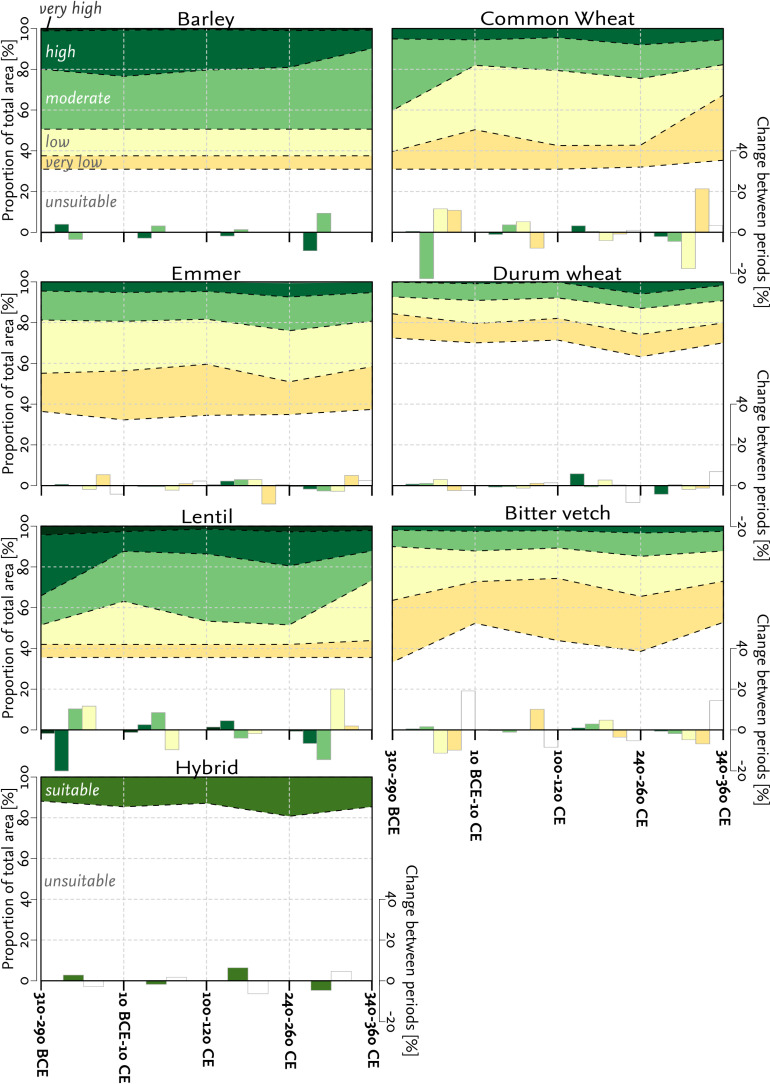
Change of the areal share of different suitability classes. Suitability classes (stacked lines) are in relation to the total area from crops combined in the hybrid model. Bar plots depict the class-specific changes in suitability between periods, showing fluctuations in cultivation suitability over time.

The topography of the western Anatolian coast is characterized by an east-west oriented graben system that built up since the Early Miocene [46]. The environs of Pergamon are shaped by the Bergama graben; the Bakırçay River, the ancient Kaikos, is the main receiving stream and flows through this graben. The micro-region exhibits a mix of sedimentary, metamorphic, and igneous rock formations, contributing to a heterogeneous landscape. The Karadağ, Kozak, Madradağ, and Yüntdağ mountain ranges surround the Bergama graben. Elevations range from the sea level in the west to about 1800 m a.s.l. in the Madradağ mountains. The Bergama graben is alluviated by the multiple tributaries of the Bakırçay, forming shallow alluvial fans as they enter the graben and an extensive alluvial plain. Morphologically, the alluvial areas of the Bakırçay River are categorized into two distinct regions: the upper and lower Bakırçay plains. The upper Bakırçay plain is located upstream of the river’s entry into the Bergama graben, southeast of Apollonia (Fig 1). The lower Bakırçay plain refers to the alluvial area within the Bergama graben. Pergamon is situated in the center of the Bergama graben, marking the division of the lower Bakırçay plain into its western and eastern parts, which is commonly used for its morphological description.

The mid- to late Holocene landscape of the region is characterized by a polygenetic nature, primarily influenced by climatic shifts and human activities [8,47]. Sediment profiles across the Pergamon micro-region reveal several sedimentation phases with substantial changes occurring around 4 ka BP, attributed to supra-regional aridization and rapid climate events, compounded by the onset of local human activities [14,47]. Unlike other prominent graben systems of the western Anatolian coast [48], there is no evidence of a Holocene estuary in the Bergama graben that silted up after the Holocene transgression. Nevertheless, the harbor of Elaia east of the Bakırçay delta (Fig 1), which is regarded as a maritime satellite of Pergamon [49–51], bears traces of human-accelerated siltation. Here, phases of sedimentation are associated with peak periods of local land use and are particularly characterized by deforestation, increased agriculture, and the cultivation of olive trees during the Roman Imperial period [51–53].

The current geomorphological and hydrographic context of the Bakırçay catchment area is characterized by hydraulic engineering interventions [39]. In addition to the channelization and diversion of the Bakırçay in the 20th century, its eastern plain, in particular, was subjected to extensive consolidation. To create a plain that could be agriculturally used efficiently, large-scale drainage and irrigation canals were constructed, Bakırçay tributaries were channelized, small hills in the plain were removed, and hundreds of wells were built [54]. From the mid-20th century onward, several rivers were dammed to facilitate irrigation of the agricultural land [54]. Compared to ancient times, modern engineering measures have a strong influence on the present water balance, particularly dam constructions in the tributary’s headwater areas. In antiquity, the city of Pergamon was supplied with water by an extensive system of aqueducts from springs all over its hinterland [55]. The extent to which these systems influenced the agricultural use of the plain is currently unknown.

### 2.2 Social setting

Pergamon was a major superregional Hellenistic and Roman political and cultural center that continued to exist as a small town in the Byzantine and Ottoman periods before becoming a regional center in the Turkish Republic. Its ancient history is evident in the architecture, traditions, and cultural practices that are still evident in modern-day Bergama and its surrounding villages. Although the site shows traces of settlement dating back to the Middle Bronze Age, Pergamon emerged as a politically and economically independent polis by the mid-4th century BCE as part of the reign of Alexander the Great [42,56]. With the death of Alexander and the subsequent division of the empire, his retainer, Lysimachos, entrusted Philetairos with the safekeeping in Pergamon. Philetairos strategically utilized the treasury entrusted to him, which laid the foundation for establishing the Attalid dynasty, also known as the Pergamene Kingdom [42]. Under the Attalid rule, the city continued to grow and prosper, becoming one of the most important centers of the Hellenistic Period in the 2nd century BC [41,56]. Agriculture must have played an important role in the local economy of the city and its spheres of influence, testified by the local practices for olive oil and wine

production, as well as grain processing, including the use of a large-scale network of millstones and presses [30,60]. There is evidence of a large number of settlements on the Bakırçay plain and the surrounding hills [3,61], indicating extensive land use as well as a development of prosperity.

The transformation of Pergamon from a Hellenistic *polis* to a Roman Imperial *metropolis* occurred in approximately four major phases [42]. The first phase is characterized by the transformation of Pergamon into a Hellenistic city during the 3rd century BCE, with the city’s center remaining concentrated on the upper part of the city hill. The second phase paralleled the peak of Attalid power during the 2nd century BCE, marked by the integration of the largest part of the city hill into a fortification, accompanied by extensive building activities and infrastructure improvements. During the third phase (1st century BCE to 1st century CE), the city mainly developed within the fortifications of the early 2nd C BCE. At the same time, during the *Pax Romana* (after 27 BCE), the onset of military security led to a decline in fortified settlements across the Pergamon micro-region [3,61]. The fourth phase of urban development and prosperity of Pergamon captured the period of the late 1st and the 2nd century CE. This phase was characterized by urban reorganization paired with extensive urban expansion towards the Bakırçay plain, along with a large building program, including prestigious buildings such as the Sanctuary of the Egyptian Deities, also known as the Red Hall. Together, these interventions likely contributed to a considerable population increase. Following this last major expansion of the ancient city, a phase of stagnation ensued, as documented by the decline of residential areas on the city’s hill slopes in the 3rd century CE and a decline in building activities. In addition, the so-called “Gothic Wall” was built in the mid-3rd century CE as an extended fortification against the raiding Goths in Asia Minor [42,62].

Today, the environs of the ancient city of Pergamon support a mix of agricultural, industrial, and residential land uses (Fig 1). Agriculture, including both traditional and modern farming practices, plays an important role in the local economy, with crops like olives, wheat, cotton, and tobacco cultivated in the plains, their direct surroundings as well as intramountainous basins [63,64].

## 3 Materials and methods

We address the diachronic developments in the agricultural potential of the Pergamon micro-region by modeling the ecological niche of commonly known crops in the Aegean region. Our modeling is based on the EcoCrop approach, which provides several environmental requirements (e.g., tolerance ranges [65]) necessary for plant development. We incorporated parameters of three physical geo-factors (climate, topography, soil) into our models to spatially delineate the cultivation suitability for particular crops in our study area on a mesoscale (Fig 3). This heuristic approach accounts for natural factors and, therefore, must be understood as a natural agroclimatic potential. As such, it does not include actual human management of the landscape with modifications, such as melioration, water management, or terracing, which are understood as additional improvements to the suitability of the land for cultivation. At the same time, the natural agroclimatic potential highlights areas that likely have been agriculturally exploited or not [66]. To address the development of cultivation suitability for different crops under various climatic settings during the social transformation of the Pergamon micro-region, we incorporated the MPI-ESM 1.2 climate model [37] and statistically downscaled it to an appropriate spatial resolution of approx. 1x1 km (Fig 3).

To assess the general natural agroclimatic potential, we calculated models for six known crop types of the ancient Aegean region under five different climatic settings (Table 1). Considering the soil degrading effects of long-term monocultural cultivation, we set up a hybrid model that comprises six commonly cultivated crops. Therefore, the hybrid model provides insights into areas that are not only niche spots for individual crops but also those that are naturally suitable for crop rotation. The hybrid model comprised two legume types, commonly used as catch crops in antiquity, and four main cereal crops [68]. No permits were required for this study, which complied with all relevant regulations.

**Table 1:** Modeled crops and their regional attestation. The listed crops are known from archaeobotanical and literary sources to have been cultivated or consumed in the Aegean region. The classification of a crop’s occurrence at a specific site, whether through trade or local cultivation, is based on the assessment by Marston and Birney (2022) [59], who followed the interpretations of the original archaeobotanical reports. Sites from the Ademnes database [58,69,70] are included, although they do not provide information on the origin of the archaeobotanical remains. For site locations, see Fig 2.

Type	Name	lat. name	EC code	Krania^*^	Kompoloi^*^	Platania^*^	Düzen tepe^*^	Gordion^*^	Smyrna^**^	Troy^***^	Kalapodi^***^
**Poaceae**	Common wheat	Triticum aestivum L.	8	xx	x	xx	xx	xx		x	x
	Emmer	Triticum diococcon Schrank	755	x		x		x	xx	x	x
	Durum wheat	Triticum durum Desf.	754	xx		xx	xx			x	x
	Barley	Hordeum vulgare L.	9	xx		xx	xx		xx	x	x
**Fabaceae**	Lentil	Lens culinaris Medikus	56	xx	x	xx	x	xx	xx	x	x
	Bitter vetch	Vicia ervilia (L.) Wild.	772	xx		xx	xx	xx	xx	x	x

EC = EcoCrop

x = present (possible from cultivation or trade)

xx = present (very likely cultivated)

* = Marston and Birney (2022) [59]

** = Maltas et al. (2023) [2]

*** = Riehl et al. (1999) [70] found in the Ademnes database [58]

**** = Kroll (1993) [69] found in the Ademnes database [58]

### 3.1 Ecological Crop Requirement (EcoCrop)

EcoCrop, developed by the Food and Agriculture Organization (FAO) of the United Nations [31], is a database designed to understand the ecological niches of specific plants and crops. It includes environmental requirements and limitations for 2,568 taxa, specifying the optimal and absolute ranges of environmental factors (e.g., climatic conditions, soil characteristics) required for successful growth. EcoCrop is based on the law of the minimum by providing parameters for each crop within both absolute and optimal ranges [71]. The database is grounded in the law of the minimum, meaning that the least favorable parameter determines the overall suitability for plant growth on a given plot of land [35]. These tolerance ranges are expressed as fuzzy data, encompassing both absolute and optimal values for each parameter (see S1 File). While this approach does not account for the full variability of ancient crop performance – especially the phenotypic plasticity of premodern landraces – it ensures transparency and reproducibility.

In our study, we considered climatic (monthly mean temperature, monthly precipitation sum), topographical (slope, topographic wetness index (TWI), wind exposure, solar radiation), and soil parameters (texture, pH) as variables (Fig 3). The EcoCrop model generates a crop-specific suitability index by combining these inputs. Input parameters were categorized as either static (unchanging across time and seasons) or dynamic (varying seasonally and across time frames) [32]. Solar radiation, while quasi-static, incorporates monthly sunshine hour differences but does not account for year-to-year variations. Topographic and pedological parameters, given the spatial resolution of ca. 1x1 km, were integrated as static inputs [72].

This study aimed to create a simple yet robust multivariate model that realistically reflects naturally complex processes. To ensure replicability, we used non-commercial datasets. Topographic parameters such as solar radiation, TWI, wind exposure, and slope were derived from the TanDEM-X DEM [40,73]. Soil data (texture, pH) were integrated using the multivariate global regression model SoilGrids [74]. While the original EcoCrop database does not account for topographic factors such as slope, wetness, and wind exposure, we have incorporated these to enhance the spatial differentiation of cultivation suitability. Slope and TWI parameters were adapted from Laabs and Knitter (2021) [29], while wind exposure tolerance ranges were developed based on present-day land use in the Pergamon micro-region. Soil texture classifications (light, moderate, heavy) from the original EcoCrop database were translated into specific grain-size compositions (clay, silt, sand) to align with SoilGrids data.

The EcoCrop model was implemented using the R package “Recocrop” [32,75]. Details of the model setup and the associated code are provided in the supplement (S1 File). The suitability index for each crop was then normalized and classified into five classes, ranging from very low to very high suitability for cultivation.

### 3.2 Archaeobotanical records

We ran the EcoCrop model for different crop types commonly used as food sources during the Hellenistic to Roman Imperial periods in the eastern Mediterranean. The selection of these crops was informed by an analysis of archaeobotanical records (Table 1; Fig 2A). Specifically, we utilized the work of Marston and Birney (2022) [59], who assessed archaeobotanical datasets from the Greek coast, central Anatolia, and the Levant to identify frequently cultivated crops. Additionally, we incorporated data from the Ademnes database [58,69,70], which harmonizes archaeobotanical records across the eastern Mediterranean and Near East. The selected crops are consistently attested across multiple regional sites, including both inland and coastal settlements, suggesting broad cultural and ecological relevance.

The list includes staple cereals such as common wheat, durum wheat, emmer, and barley, which appear in nearly all archaeobotanical contexts and formed the dietary base (Table 1). Lentil and bitter vetch were included for their frequent occurrence in the archaeobotanical records, their drought tolerance, and their functional role in diversified cropping systems. However, agricultural practices in the region likely included a broader range of crops to enhance the resilience of food supplies. Other crops (e.g., grass pea, chickpea, spelt, millet) were excluded from the final model due to sparse attestation, limited regional relevance, or uncertain cultivation status during the study period. However, the integration of additional crops during model development did not result in further analytical benefit and was therefore not pursued in the final model.

In the case of barley, the EcoCrop database provides generalized climatic thresholds for the species level and does not distinguish between specific subspecies or varieties, such as 2-row or 6-row, hulled or naked barley. While archaeobotanical evidence suggests that 6-row hulled barley was commonly cultivated as food in western Anatolia [76], our model reflects the broader species-level parameters.

The physician Galen mentions wheat, emmer, and einkorn and vegetables such as lettuce consumed in Pergamon [30] (cf. Gal. 6, 518). While not all food sources can be reconstructed from physical remains, archaeobotanical studies across the Mediterranean offer valuable insights into crops that were likely cultivated. Pollen records from the coastal city of Elaia indicate a consistent presence of cereal pollen between 4 ka and 1 ka BP [52], although they cannot distinguish specific crop types. Similarly, the Kara Göl, a mountain lake located in the Kara Dağı southwest of Pergamon (Fig 1), includes records of cereal pollen [77]. However, its low temporal resolution makes it unsuitable for detailed comparison.

While the selected crops (Table 1) are consistently attested across the broader eastern Mediterranean region during the Hellenistic and Roman periods, recent work by Marston and Castellano (2023) [78] highlights that the importance and distribution of individual crops likely shifted over time. Similarly, Douché et al. (2021) [79] provide a synthesis of archaeobotanical records from first-millennium BCE sites in Greece, confirming the continued presence of staple cereals and pulses – such as emmer, barley, and lentil – at both inland and coastal sites. However, in the absence of sufficient high-resolution archaeobotanical evidence for the Pergamon micro-region, this study adopts a temporally averaged perspective on crop use.

Additionally, perennial crops such as olive, grape, and fig – although central to the ancient Mediterranean economy – were not included in the model due to their multi-year life cycle, differing agroecological requirements, and dependence on more intensive or managed systems (e.g., terracing, pruning, irrigation). Since the present study focuses on the climatic potential for rainfed annual field crops, perennial species should be considered in future approaches.

### 3.3 Climate data

To assess the paleoclimatic history along the Turkish Aegean coast, we utilized a transient paleo simulation [80] conducted with the Max Planck Institute Earth System Model version 1.2 (MPI-ESM1.2) [37]. The simulation results were downscaled using WorldClim 2.1 [81], a modern dataset based on station and satellite observations with a spatial resolution of about 1 × 1 km. Validation was performed using an updated version of the LegacyPollen 2.0 dataset [57], which provides pollen-based climate reconstructions from a globally harmonized dataset.

Five snapshots were selected for downscaling (see section 3.3.3). Focusing on snapshots helped reduce processing time and storage requirements. These snapshots were selected to align with historically important periods, providing a contextualized understanding of modeled climatic changes within broader phases of climate variability (Fig 2). Each snapshot represents a 20-year interval, including monthly average temperature and precipitation data. While the standard climate normal period spans 30 years, we adopted a 20-year interval to reflect better the temporal scales relevant to agricultural adaptation and human perception of climate change. Modern decision-making for annual and perennial crop systems typically occurs over 5–10 years [82]. However, given the generational nature of ancient agricultural traditions [83], a 20-year period was considered an appropriate compromise for this study.

#### 3.3.1 MPI-ESM 1.2.

A transient climate simulation [80] within the Max Planck Institute Earth System model version 1.2 (MPI-ESM 1.2) [37] has been used as the basis for the paleoclimate assessment. This simulation spans approximately 8000 years (6999 BCE to 1850 CE). The model is forced by orbital-induced insolation, greenhouse gas concentration, stratospheric aerosol injections imitating volcanic eruptions, and spectral solar irradiance changes while considering transitional land use over the last 2000 years. The MPI-ESM 1.2 was computed for a T63 Grid (1.875° x 1.865°, ca. 200 x 160 km at 39°N), representing a high spatial resolution for paleoclimatic simulations. This simulation, along with a slightly different one in the same model, has already been successfully used to shed light on prominent climatic-cultural changes in the Holocene, such as the end of the African humid period [84] or mid-Holocene climatic Influences on settlement dynamics in central Iran [85]. The model simulates temporal variability on different time scales, related to the prescribed forcing or the internal variability of the simulated climate system. Thus, single events caused by factors beyond the given forcing cannot be reproduced by the model. Similarly, climatic events related to internal variability may not occur simultaneously in the simulation, as observed in proxy archives.

#### 3.3.2 WorldClim 2.1.

The WorldClim 2.1 dataset [81] offers high-resolution climate data (30 arc seconds; ca. 0.72 km at 39°N) on a global scale, covering the period 1970–2000 with monthly and annual averages. It includes key climate variables, such as temperature and precipitation, as well as other bioclimatic variables. Data processing involved interpolation of thin-plate splines to generate continuous raster grids, ensuring rigorous quality control through outlier detection and validation against ground truth using data from up to 60 000 climate stations [81]. Widely used in ecological and environmental research, it supports studies like species distribution modeling, habitat suitability assessments, biodiversity analyses, and climate change impact assessments [74,86,87], despite limitations such as spatial interpolation errors and biases due to uneven station distribution [81]. Nevertheless, due to its high spatial resolution, the dataset can account for orographic gradients of climate variables.

Besides the downscaling of the MPI-ESM 1.2 data, we used the WorldClim 2.1 dataset to calculate a “Climate Similarity Index” (Fig 2) by applying the “calc_similarity” function from the analogues package in R (https://github.com/CIAT-DAPA/analogues). This index was used to identify suitable reference sites within the updated pollen-based climate reconstruction dataset for validating the climate data (see section 3.3.4).

#### 3.3.3 Downscaling.

The MPI-ESM 1.2 simulation provides a temporally high-resolution option for understanding large-scale dynamic climate systems; however, the spatial resolution of 1.875 x 1.865 degrees, which is high for global paleoclimate simulations, poses a challenge for the mesoscale differentiation of topographic complexity in climate [85]. This limitation poses a challenge as the horizontal and vertical distribution of temperature and precipitation is critical for determining the suitability of specific crops for cultivation [81]. Statistical downscaling was necessary to integrate the high spatio-temporal resolution of the MPI-ESM 1.2 simulation into our agricultural potential model. We applied the delta method, using contemporary climate data from WorldClim 2.1 as a reference point, to enhance the spatial resolution of the MPI-ESM 1.2 outputs [88]. The delta method involves pixel-wise differentiation between simulated and reference data: the difference between the two datasets is added to the simulation outputs, effectively downscaling the climate variables [88]. To minimize edge effects, the MPI-ESM 1.2 data were first globally interpolated and then adjusted to match the spatial resolution of WorldClim 2.1.

#### 3.3.4 Pollen-based climate reconstructions.

We leveraged pollen-based climate reconstruction from the LegacyClimate 1.0 dataset [89] to evaluate paleoclimate simulations from the MPI-ESM 1.2. The data of pollen-based reconstructions was extended by some further sites included in the harmonized pollen dataset of LegacyPollen 2.0 [57] mostly based on records stored in the Neotoma database [90].

For our study, nine records from a region around the Aegean Sea were selected based on their climate similarity to Pergamon (>70% - Fig 2A, see section 3.3.2), as well as the number of radiocarbon dates between 400 BCE and 400 CE (n > 5). The lake sites of Beyşehir and Lerna included two records, both of which have been incorporated into the overall validation [90].

Reconstructed temperature and precipitation values were linearly interpolated for the time frame between 400 BCE and 400 CE.

#### 3.3.5 Comparison of pollen-based climate reconstructions and MPI-ESM 1.2 simulations.

To evaluate the reliability of our dynamic climatic input parameters (temperature and precipitation), we compared our forcing-based climate data from the MPI-ESM 1.2 [37] for the late Holocene and the pollen-based climate reconstruction from the LegacyPollen 2.0 data set [57]. Given the location of Pergamon at the intersection of four adjacent cells within the MPI- ESM 1.2 dataset, and to prevent an imbalance in favor of a single cell in a global model, the climate variables from the four surrounding cells were averaged for comparison (see Fig 2).

Due to the different prerequisites of the forcing-based MPI-ESM 1.2 model and the pollen-based reconstructions, a comparison in a Euclidean space would not account for a potential time lag between climate variability and the ecological response. To account for these slow processes, simulated climate and pollen-based reconstructions are compared and validated by a dynamic-time-warping algorithm (dtw) using the eponymous R-package [91]. The dtw-algorithm can identify similarities between multiple time series by comparing several points in time instead of one-to-one comparisons in Euclidean space. The standardized z-scores were compared using Rabiner-Juang step patterns type “VI-c” in the dtw R-package [91] (Fig 4B).

Overall, the temperature and precipitation values from both reconstructions reveal a high level of agreement (Fig 4B). The alignment path in the dtw analysis is nearly linear but not perfectly diagonal, indicating a general agreement between the two time series with some phase shifts and time lags. The relatively close alignment of the paths to the diagonal suggests that the two reconstructions generally capture similar patterns, although not identical. The time lags between the two models are primarily evident in the precipitation values (Fig 4B). The normalized distance between the precipitation values from the model simulation and the pollen-based reconstruction is 79 mm, with the reconstructed values exhibiting a higher mean than those from the MPI-ESM simulation. The standard deviation of the reconstructed MAT precipitation values is approximately 130–250 mm, while that of the MPI-ESM simulation is approximately 80 mm. The estimated distance between the two reconstructions is less than the actual error, indicating that the almost aligned path between the two time series is a valid representation of the agreement. The temperature values of both time series exhibit a better alignment (Fig 4B). However, the normalized distance between the two reconstructions is approximately 5°C, with pollen-based reconstruction data showing a lower value than the MPI-ESM estimations. Here, the standard deviation of the reconstructed mean annual temperature amounts between 1.8°C and 3.8°C. In comparison, the standard deviation of the MPI-ESM simulation is approximately 2.9°C, which is lower than the distance between both reconstructions. Overall, when considering the standard deviation between both reconstructions, the simulated values overlap, which should be understood as a valid agreement.

#### 3.3.6 SoilGrids.

In our model, we used present-day soil parameters (SoilGrids [74]) to reconstruct the agroclimatic potential of Pergamon and its environs. Physical soil characteristics, such as texture and pH, tend to evolve at a relatively slow pace unless altered by human activities, climate shifts, or events [92,93]. While alluvial plains change due to sediment deposition, soil properties such as texture and pH change relatively slowly unless subjected to major events. Due to our model’s coarse spatial resolution (~1x1 km grid), localized variability from geomorphic processes can be assumed to be averaged out, allowing regional soil trends to dominate.

To evaluate the soil data used in this study, the SoilGrids texture and pH data for the study area [74] were compared with topsoil sample data collected from the environs of Pergamon [67]. The comparison revealed high Root Mean Square Errors (RMSE) for sand (46%), silt (26%), clay (31%), and pH (25%). These discrepancies can be attributed to differences in spatial resolution and measurement methodologies between the datasets. Additionally, the high heterogeneity of the catchment area and the limited number of sampling points in the field data likely contributed to the observed deviations.

Errors inherent in the SoilGrids dataset, such as those resulting from interpolation and extrapolation processes, as well as potential inaccuracies during the initial data collection and processing phases, further compounded the differences [74]. However, the EcoCrop model employed in this study utilizes a fuzzy data approach with absolute and optimal tolerance ranges. This methodological design minimizes the impact of such discrepancies, as it does not depend on precise input values but rather accommodates variability within defined ranges. As a result, the model enables more robust predictions of agricultural suitability despite the uncertainties inherent in the soil texture and pH data. This approach ensures that the intrinsic variability and methodological differences between datasets do not overly compromise the accuracy of the agricultural suitability assessments.

## 4 Results

### 4.1 Climate variation in western Anatolia

Between 400 BCE and 400 CE, the climate in western Anatolia, as represented by four MPI-ESM 1.2 grid cells (Fig 2A), exhibited variability in temperature and precipitation (Figs 2B and 4A). The MPI-ESM simulation and the pollen-based reconstruction both highlighted alternating phases of warmer and drier conditions, as well as colder and wetter conditions. However, the exact timing and intensity of these phases varied between both. These climatic phases vary in length, with major phases lasting up to 60 years and minor phases persisting for 10–30 years.

The climatic states derived from the MPI-ESM simulation used as inputs for the agricultural model were based on the five snapshots (310–290 BCE, 10 BCE – 10 CE, 100–120 CE, 240–260 CE, and 340–360 CE; Fig 2b) representing different climatic states within the study period. The snapshots reflected the variability within the dataset rather than precise historical conditions. For example, wetter and colder conditions were more prominent in the earlier snapshots, while later snapshots tended to represent warmer and drier scenarios. Both models indicated substantial variability even within these generalized phases, with some snapshots capturing transitional states between broader trends (Figs 2 and 4A). These results emphasized the complexity of climatic variability in western Anatolia during the study period.

The first snapshot, corresponding to the period from 310 to 290 BCE, represents a time marked by colder and wetter conditions, as confirmed by the pollen-based reconstruction. Precipitation values were generally above average, while temperature values were below average, indicating a wetter and cooler phase relative to the overall variability of the study period.

The second snapshot, from 10 BCE to 10 CE, reflects a transitional phase. Temperature values in both datasets – the MPI-ESM simulation and the pollen-based reconstruction – were closer to or slightly above average, while precipitation displayed moderate variability. This snapshot captures conditions of relative balance between warmer and drier tendencies, as well as wetter and colder intervals.

The third snapshot, spanning 100–120 CE, represents a particularly warm phase, with temperature values consistently above average in both the MPI-ESM simulation and the pollen-based reconstruction. Precipitation, while variable, remained generally close to average, indicating a predominantly warm and stable drier climate state during this interval.

The fourth snapshot, corresponding to the period from 240 to 260 CE, captures a transitional phase characterized by above-average but slightly decreasing temperatures and precipitation. The snapshot indicates somewhat cooler and wetter conditions compared to the preceding snapshot, reflecting a shift toward a more moderate climate state.

The fifth snapshot, spanning 340–360 CE, represents a cooler setting than observed in the fourth snapshot. While temperature remained above average, precipitation values were close to or slightly below average in the MPI-ESM simulation. However, the pollen-based reconstruction reflected a wider difference between warmer temperatures and below-average precipitation.

These snapshots, incorporating 20-year averages, served as the climatic forcings for the agricultural model, providing a framework for assessing potential cultivation conditions under varying temperature and precipitation regimes.

### 4.2 Crop-Specific suitability for cultivation

The spatial statistics refer to the Pergamon micro-region, which is defined by the combined boundaries of the walking distance and the watersheds of its two main rivers (Fig 1). The crop-specific models indicate a moderate to high suitability of areas in the alluvial plains of the Bergama graben for agricultural cultivation across all analyzed snapshots.

The areas showing the highest modeled suitability for cultivation are in the coastal region surrounding the Gulf of Çandarlı and the Gulf of Dikili. The coastal regions exhibited only minor temporal variations in cultivation suitability (Fig 5). In contrast, some areas in the western lower plain (between Atarneus and Elaia) and the eastern lower Bakırçay plain (approximately 5 km east of Pergamon) were classified as unsuitable for cultivation across all time slices. High topographic wetness primarily characterizes these areas compared to other parts of the Bakırçay plain; in our model, topographic wetness is a temporally static parameter (see S1 File). In contrast to some of the crop-specific models, the hybrid model does not indicate any cultivation suitability on the Kozak plateau (approximately 20 km north of Pergamon) or in any other highlands (Fig 5).

#### 4.2.1 Snapshot 1 - 310-290 BCE.

During this period, the foothills and piedmont areas flanking the Bergama graben, specifically the transition zone between the highland and floodplain, were more suitable for cultivating several crops than the Bakırçay floodplain areas (Fig 5). This pattern is apparent for almost all crops of the analyzed time slices except for common wheat. During this snapshot, the western and eastern plains, as well as the foothill and piedmont zones, are universally suitable for cultivating common wheat. Across all crop models for this snapshot, vast areas in the mountainous parts of the Pergamon micro-region were classified as unsuitable for cultivation. Only the Kozak plateau, situated within the Kozak mountains (Fig 1), exhibited high variability in cultivation suitability across the time frames, shifting from very low to high. In the elevated plateau areas, barley cultivation is most suitable (Fig 5). The hybrid model indicates general suitability for cultivating the most common crops along the coastal areas and the western lower Bakırçay plain. Within the plain, the suitability of hybrid cultivation slightly exceeded that of the alluvial fan at Pergamon.

#### 4.2.2 Snapshot 2 - 10 BCE-10 CE.

During this period, cultivation suitability extended towards the eastern lower Bakırçay plain compared to snapshot 1 (Fig 5). The suitability of the highlands adjacent to the eastern lower Bakırçy plain for the cultivation of common wheat decreased and concentrated in the flat areas of the plain.

Areas that were moderately suitable for wheat cultivation were most affected by climate alterations at this stage compared to other crops or legumes, with a reduction in the modeled possible cultivation area of more than 20%pt. (Fig 6). Accordingly, there was an increase in areas with low to very low suitability for wheat. Compared to snapshot 1, the other *Triticum* species showed only minor changes in areas suitable for cultivation. The suitability of barley cultivation was only slightly affected, specifically in a decrease in the proportion of areas on the Kozak plateau characterized by high cultivation suitability and an increase in areas with suitable cultivation in the northeast of the study area (Fig 5). The suitability for cultivating legumes also decreased in the higher elevations of the Kozak plateau during this period. The model showed no suitability for cultivating bitter vetch and a decrease in cultivation suitability from high to moderate for lentils on the Kozak plateau.

The proportion of areas unsuitable for cultivating bitter vetch increased by almost 20%pt., while areas of high suitability for lentils decreased by a similar share (Fig 6). The hybrid model indicated small expansions of areas suitable for cultivation with catch crops (+3%pt.) in the northern and southern piedmonts of the eastern plain, without changes in the highlands.

#### 4.2.3 Snapshot 3 - 100-120 CE.

For the third snapshot, our models indicate an increase in suitability for wheat and lentil cultivation during warmer and drier conditions (Fig 2). Compared to the second snapshot, there was a decrease in areas classified as very low suitable for wheat cultivation and an increase in areas classified as low to moderately suitable (Fig 6). For common wheat, these changes occurred mainly in the eastern part of the Bergama graben, with a wide increase in cultivation suitability in the hilly areas between Pergamon and Allianoi (Fig 5). In addition, areas suitable for lentil cultivation showed a rise of about 10%pt. of areas with medium cultivation suitability and a decrease of approximately 10%pt. of areas with low cultivation suitability (Fig 6). For the remaining crops, cultivation suitability showed only minor changes, remaining largely stable. However, despite the changes in cultivation suitability for wheat and lentils, the hybrid model indicated a slight decrease in cultivation suitability compared to the second snapshot. The reduction in hybrid cultivation suitability (−2%pt.) occurred predominantly in the northern and southern parts of the eastern lower Bakırçay plain.

#### 4.2.4 Snapshot 4 - 240-260 CE.

For the fourth snapshot, the hybrid model indicated an increase in cultivation suitability (+6%pt.) for different crop types compared to the third snapshot (Fig 6), with the piedmont areas on the eastern edge of the Bergama graben showing the increase most distinctly (Fig 5). These changes coincided with increased suitability for cultivating bitter vetch and barley in the Kozak plateau. Additionally, the area suitable for cultivating durum wheat expanded widely towards the eastern part of the alluvial plain. The suitability for the cultivation of emmer increases with the proportion of areas characterized by being high, moderate, and low suitable for emmer cultivation increases, whereas the proportion of areas of very low suitability accordingly decreased (Fig 6); these changes can be mainly observed in the foothills between Pergamon and Allianoi.

#### 4.2.5 Snapshot 5 - 340-360 CE.

For the fifth snapshot, the hybrid model indicated a reduction in areas suitable for agriculture of about 5%pt. compared to the fourth snapshot (Fig 6). These changes in the hybrid model were observed particularly at the eastern edge of the eastern lower Bakırçay plain and in the hilly areas between Pergamon and Allianoi (Fig 5). In parallel, the model outcomes for the cultivation suitability of common wheat indicated an increase of more than 20%pt. of very suitable areas, while the number of suitable areas decreased from low to high. In contrast, the suitability for cultivating common wheat decreased, particularly in the piedmont zone of the Bergama graben. Areas of moderate to high suitability for lentil cultivation on the Kozak plateau, as well as in the foothills between Pergamon and Allianoi, in the fourth snapshot, turned out to be less suitable. The proportion of areas suitable for cultivating bitter vetch decreased strongly, with 17% of the total area now classified as unsuitable (compared to the previous snapshot). This has reached approximately the same level of expansion as at the turn of the millennium (second snapshot) and can primarily be observed on the Kozak plateau. Cultivation suitability for barley, which remained constant in previous snapshots, also showed a decline in previously highly suitable areas east of the study area.

#### 4.2.6 Variability of cultivation suitability across snapshots.

The coefficient of variation across the five snapshots provides insights into which crops and areas are most susceptible to climatic fluctuations in terms of cultivation suitability. Barley and lentils exhibited the lowest variability in suitability across the analyzed snapshots (Fig 6). In particular, the Kozak plateau and the eastern parts of the Pergamon micro-region showed minimal variability in suitability for lentil cultivation. In contrast, these same areas experienced extensive fluctuations in the suitability for common wheat, emmer, and bitter vetch. Notably, the suitability bitter vetch varied on the Kozak plateau (Fig 5), while common wheat and emmer displayed pronounced variability both on the plateau and in the hills between Pergamon and Allianoi. The durum wheat suitability, by contrast, showed fluctuations in the piedmont zone of the Bergama graben and the eastern lower Bakırçay plain. However, the lower Bakırçay plain as a whole exhibited relatively low variability. These spatial and crop-specific differences reflect the varying climatic sensitivity of individual taxa, as determined by their environmental tolerance parameters (see Supplement S1 File).

The hybrid cultivation suitability model mirrored the pattern observed for durum wheat, with changes in suitability concentrated along the piedmont zone of the Bergama graben. Regarding the climate variables, the coefficient of change for both the annual sum of precipitation and the mean annual temperature (Figs 8A, B) indicates that coastal areas – particularly in the vicinity of Elaia and Atarneus – exhibited the greatest variability in precipitation (up to 79%) across the five snapshots, compared to the more stable conditions in continental hinterland. In contrast, the eastern and northern mountainous areas of the Pergamon micro-region showed the highest variation in temperature, reaching up to 65%.

**Fig 7:**
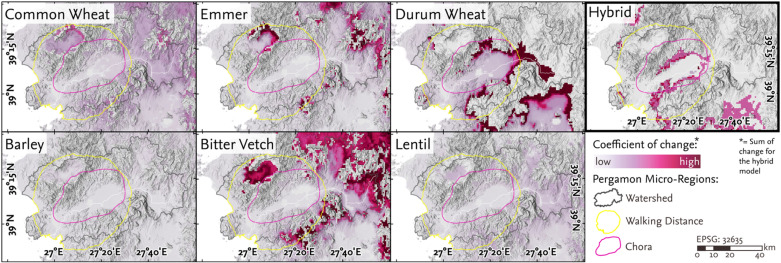
Change in cultivation suitability across the seven common crops and the integrated hybrid model. Change is expressed as a coefficient of variation across all five snapshots (Fig 5). The change in the binary hybrid model is described as the sum of changes across all snapshots.

**Fig 8:**
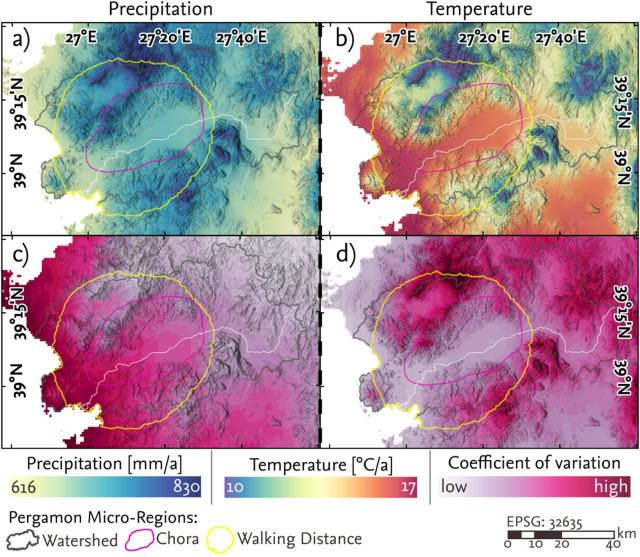
Spatial distribution of downscaled average climate variables across the five snapshots (310-290 BCE, 10 BCE-10 CE, 100-120 CE, 240-260 CE, 340-360 CE) a) average annual sum of precipitation b) average annual temperature c) coefficient of variation of the average annual sum of precipitation across the five time periods d) coefficient of variation of the average annual temperature across the five time periods.

## 5 Discussion

The dynamics of agricultural potential in the Pergamon micro-region during antiquity were assessed by integrating downscaled agroclimatic variables derived from the MPI-ESM 1.2 hindcast climate simulation into an ecological niche modeling framework (Ecocrop). The primary objective was to analyze the temporal and spatial variability of crop-specific agricultural suitability from 400 BCE to 400 CE, a period marked by profound socio-ecological transformations. The modeled results provide a basis for understanding the influence of climatic variability on agricultural practices. However, these results should be viewed as indicative of possible scenarios rather than definitive reconstructions of historical realities. The inherent variability in the MPI-ESM 1.2 simulation reflects potential climatic states, enabling an analysis of how such conditions may have influenced agriculture and socio-economic systems in the region. Generally, the simulations indicate that rain-fed agriculture in the vicinity of the Pergamon micro-region was subject to climatic fluctuations, which consequently must have influenced human perceptions of cultivation potential and, in turn, land use [94].

Numerous pollen [90,95] and archaeobotanical [58] records exist from the eastern Mediterranean region; however, the majority of these records are too unspecific in their classification or show a too low temporal resolution to compare them with our models. Close records can, nonetheless, at least give a rough estimate of agricultural dynamics as well as adaption processes in the region. The closest pollen records are located in the western part of the Pergamon micro-region, in the vicinity of the harbor city of Elaia (Fig 1; [52]), providing valuable paleoenvironmental data that contextualized our modeled results. The record consistently showed the presence of cereal pollen between 4 ka and 1 ka BP, reflecting long-term agricultural activity. Peaks in cereal pollen concentrations around 2.4 ka BP and 2.1 ka BP suggested phases of agricultural intensification. Although the 2.4 ka BP peak fell outside the timeframe of this study, the 2.1 ka BP peak overlapped with the first modeled snapshot (310–290 BCE). However, this intensification does not coincide with the limited agricultural suitability reflected in our model, which shows a period of colder and wetter conditions. From approximately 2 ka BP onward, cereal pollen concentrations declined, reaching a minimum at around 1.8 ka BP. This reduction corresponded with the third snapshot (100–120 CE) of the hybrid model, which indicated a decline in suitability, particularly for emmer. Crops were likely affected by the higher temperatures and lower-than-average precipitation simulated during this period. Conversely, the fourth snapshot (240–260 CE) represented a moderate climatic state, characterized by slightly above-average temperatures and near-average precipitation, which corresponded to the highest extent of agricultural suitability in the model. This peak aligned with a resurgence in cereal pollen concentrations in the Elaia record [52], suggesting favorable conditions for agricultural activity.

While there is no direct connection between the peaks in the pollen record and the modeled suitability due to the delayed ecological feedback and sedimentation rates, the alignment of broader trends underscores the plausibility of the modeled scenarios. The agreement between the Elaia pollen record and the modeled outputs supported the validity of the MPI-ESM 1.2-derived climatic forcing despite acknowledging the inherent uncertainties in temporal alignment and spatial resolution. Similarly, the pollen dynamics recorded during this period indicate a constantly changing landscape, representing a dynamic socio-ecological system.

### 5.1 Spatio-temporal dynamics and their implications

The hybrid cultivation suitability model revealed that the areas with the highest suitability for cultivation between 400 BCE and 400 CE were concentrated in the coastal plains around Atarneus and Elaia, as well as large parts of the western lower Bakırçay plain (Fig 5). In contrast, the eastern lower plain exhibited more variable suitability, being cultivable only during certain snapshots. This variability reflects climatic and environmental gradients, as the western plain was characterized by higher average temperatures and lower annual precipitation. In contrast, despite similarly warm conditions, the eastern plain experienced greater humidity (Fig 8).

Our findings diverged from previous studies of cultivation suitability in the Pergamon micro-region, such as those by Laabs and Knitter (2021) [29], which suggested high arability across the entire lower Bakırçay plain. A key reason for this discrepancy is their reliance on static topographic parameters – such as slope, aspect, and topographic wetness – as primary indicators of agricultural suitability. Our results, incorporating both climatic and topographic data, demonstrated a west-to-east gradient in temperature and precipitation, with the eastern plain less suitable for the continuous cultivation of multiple crops. Today’s land use patterns and suitability classifications, as noted by Laabs and Knitter (2021) [29], equally characterize the western and the eastern lower Bakırçay plain as agriculturally suitable areas. However, this does not necessarily reflect historical conditions, as the Bakırçay alluvial plain and the coastal areas of the Gulf of Çandarlı and Gulf of Dikili have been extensively modified by irrigation and drainage systems. Historical travel reports, such as those by Cockerell (1810–1817) [96], describe extensive waterlogging in the plains during the rainy winter months, necessitating the construction of elevated causeways. Today, a network of drainage ditches mitigates this risk of waterlogging. In our model, areas identified as unsuitable for cultivation corresponded to zones of high topographic wetness, suggesting they were historically prone to waterlogging (see S1 File).

Despite these environmental challenges, ancient accounts by scholars such as Strabo and Galen described the Kaikos (Bakırçay) floodplain as a fertile landscape [30,56,97]. Seasonal flooding brought fine, mineral-rich sediments into the plain, creating vertical accumulations that supported agricultural activities [30]. Early 20th-century mapping by Philippson (scale 1:300.000 [97]) indicated that the Bakırçay meandered with low sinuosity, traversing the plain centrally. These hydrological dynamics likely required adaptation to periodic flooding, reinforcing the view that the western Bakırçay plain was more fertile and intensively cultivated compared to the eastern plain, where livestock farming may have been more prominent [30].

The high cultivation suitability of the western lower plain aligns with settlement findings from archaeological surveys across the western Bakırçay plain and its adjacent foothills [3]. Numerous Hellenistic and Roman settlements, identified as farmsteads or hamlets, were located near major road networks that crossed the alluvial plain [3,98,99]. Spatial analysis revealed shifts in settlement patterns between the Hellenistic and Roman periods [61]. While Hellenistic settlements were often located on slopes, ridges, and hilltops, Roman settlements increasingly occupied piedmont areas and, to a lesser extent, the alluvial plain itself [3,61]. This shift suggests a closer connection between Roman settlements and agricultural activities on the plain. In contrast, the eastern lower Bakırçay plain has not been extensively analyzed archaeologically yet, precluding a direct comparison between ancient settlement density and modeled suitability for cultivation. However, farmsteads and hamlets similar to those on the western plain have been documented in the north-eastern hinterland of Pergamon, in the hilly intramontane areas between Pergamon and Allianoi [3]. These elevated areas adjoin the eastern plain and were identified as increasingly suitable for cultivation in the hybrid model. The eastern expansion of agricultural suitability reached its greatest extent in the fourth snapshot (240–260 CE), encompassing much of the eastern plain. The results suggest that while the lower eastern Bakırçay plain may have been agriculturally favorable, it likely supported a lower diversity of crops compared to the western plain. By contrast, the foothills between Pergamon and Allianoi exhibited well-balanced cultivation suitability (Fig 5), making them potentially important for diversified agricultural practices.

### 5.2 Agroclimatic potential and settlement activities

The hybrid cultivation suitability model highlights distinct spatial patterns of agricultural potential in the Pergamon micro-region across five modeled climatic states. These states, represented by snapshots derived from the MPI-ESM 1.2 simulation, reflect potential conditions rather than precise reconstructions of historical realities. However, they provide a useful framework for exploring how varying climatic conditions might have influenced agricultural practices and socio-economic developments in the region.

Across all snapshots, the western Bakırçay plain, including the alluvial fan of the Selinos River, consistently exhibited high agricultural suitability. These areas likely offered stable conditions for cultivation under most scenarios, aligning with the role of fertile plains as the foundation of agricultural systems. In contrast, the eastern Bakırçay plain and the Kozak plateau exhibited greater variability, suggesting that their agricultural potential was more sensitive to climatic shifts and required adaptive strategies to optimize land use.

These dynamics are also reflected in the modeled suitability of individual crops. While cereals such as barley and common wheat consistently perform well in the western plain, bitter vetch – a crop known for its resilience and long cultivation history [70,76] - appears only marginally suitable in some snapshots. The EcoCrop model emphasizes optimal yield conditions based on modern climatic thresholds [35], which may underestimate the crop’s tolerance to marginal environments historically exploited through low-input or flexible cultivation strategies. Additionally, the crop parameters used in the EcoCrop model are based on modern cultivars, which may differ from ancient crops in their tolerance to environmental stress, growth periods, and yield stability. As Heinrich and Hansen (2021) [15] have emphasized, locally adapted ancient crop varieties likely exhibited a broader phenotypic plasticity than modern commercial strains. While the use of modern performance data remains the best available option in the absence of ancient physiological datasets, it introduces a degree of uncertainty that must be considered when interpreting suitability scores. These outcomes highlight the heuristic role of the model in identifying interpretive tensions between ecological niche modeling and archaeobotanical evidence. Our goal was not to reconstruct precise historical cultivation practices, but to assess how even moderate climatic fluctuations within species-level tolerance ranges could have impacted agroclimatic suitability and, by extension, socio-ecological resilience.

#### 5.2.1 Hellenistic Period (310–290 BCE, Snapshot 1).

The first snapshot reflects a colder and wetter climatic state, with suitability concentrated in the western part of the micro-region. These conditions align with broader patterns of agricultural intensification and territorial organization observed across coastal Anatolia and Greece during the early Hellenistic period [100,101]. If such conditions prevailed, agrarian practices in Pergamon may have been localized around the most fertile areas [101], such as the Selinos alluvial fan and the western Bakırçay plain, supporting a predominantly self-sufficient agrarian economy. While the exact historical alignment is uncertain, this climatic state could have provided the environmental foundation for the establishment of rural farmsteads typical of the Hellenistic world [100]. Population growth, coupled with the necessity for additional arable land, gave rise to alterations in agricultural practices and settlement structures throughout the Aegean region [100,101]. Similarly, in Pergamon, the focus on securing suitable land for crop production likely followed this regional trend of optimizing agricultural output to support a growing urban population from the 2nd century BC onwards [42]. Rural landscapes across the Hellenistic world often centered on self-sufficient farmsteads that supported both local consumption and, in some cases, external trade [100].

#### 5.2.2 Transition to Roman Rule (10 BCE–10 CE, Snapshot 2).

The second snapshot represents a transitional climate characterized by near-average temperatures and moderate precipitation variability. Under such conditions, agricultural suitability extended further eastward into the Bakırçay plain, potentially allowing for diversification in crop choices and the utilization of formerly less fertile areas. This scenario hypothetically aligns with the period of political stability provided by the *Pax Romana*, during which previously marginal lands may have been brought under cultivation [61]. Considering the similarity between the pollen-based climate reconstruction and the MPI-ESM simulation in terms of an above-average temperature plateau and precipitation variations (Fig 4), the dynamics captured in the second snapshot might be closely related to actual forcing on land use. If these conditions coincided with Roman reorganization, they could have supported the agricultural expansion necessary to sustain a growing urban population and increased regional integration. This process aligns with the expanding agricultural potential in remote areas, such as the Kozak plateau, for barley production in the hybrid model (Fig 5), which would have been essential for settlements like Perperene in the elevated hinterland of Pergamon [20]. However, about Perperene, there are only the descriptions of viticulture provided by Galen for this particular region [30] (cf. Gal. 15, 645, 9-10; Gal. 12, 102). From archaeological evidence, we know that with increasing political stability, societal organization and long-term planning became less focused on protection, resulting in a shift in the social metabolism towards resource consumption as well as an increasing settlement in more open relief, such as foot slopes [8,61], which were suitable agricultural areas according to our model. However, contrary to the assumption of a stable warm-wet period, assumed by the “Roman” Climate Optimum [26,27], the simulation data suggested ongoing climatic changes, which, while being under the influence of a political reorganization, must have been challenging for the actual planning of the agrarian system [102]. Similar patterns of agricultural intensification under moderate climatic regimes have been observed in other parts of Anatolia, where political stability during the Roman period enabled shifts toward surplus production and integration into broader trade networks [103,104]. However, such expansions were also linked to fiscal pressures and surplus extraction, suggesting that land-use decisions were driven not solely by climate variability but also by administrative and economic restructuring [103].

#### 5.2.3 Trajanic-Hadrianic Era (100–120 CE, Snapshot 3).

The third snapshot reflects a particularly warm climatic state characterized by below-average precipitation. This period overlaps with Pergamon’s peak in population and urban expansion during the Trajanic-Hadrianic era [42]. Hypothetically, the reduced agricultural suitability indicated by the model may have posed challenges for local food production, potentially requiring Pergamon to rely more heavily on Roman trade networks to supplement its food supply. If such climatic conditions prevailed, they could also have influenced shifts in land use and resource allocation, with increased demands on forestry and construction potentially leading to higher environmental pressures. Considering both the increasing demand for food and the growing need for labor-intensive activities such as forestry, silviculture, construction, craftsmanship, and logistics [29], there may have been a considerable deficit in the supply of agricultural products. Such a paradox could indicate a shift from local self-sufficiency towards a greater reliance on the Roman trade networks, which may have enabled Pergamon and its micro-region to sustain themselves despite challenges in local agricultural productivity [105–107]. Alongside these pressures, changing landholding patterns - such as the consolidation of estates or abandonment of marginal lands [61] - likely also shaped regional food security. In this context, Rosenstein (2008) [108] argues that already during the Middle and Late Roman Republic, landowners did not necessarily pursue intensive, profit-maximizing agriculture. Instead, estates were often held for social prestige and long-term security and frequently leased to tenants or managed indirectly [108]. If similar dynamics applied in Asia Minor, they may have further contributed to limited local food production.

Furthermore, this period also marked a peak in sediment dynamics, likely due to intensified human activities, including forest thinning or even deforestation, agricultural expansion, and construction [14,109]. Increased land utilization has likely led to higher soil erosion rates and alterations to the local hydrological system, thereby exerting additional pressure on the region’s agricultural systems [12]. When considered alongside fluctuating climate conditions, these environmental pressures suggest that while the urban center flourished, the surrounding landscape faced considerable challenges. This tension between human activity and environmental constraints remains visible today as agriculture in the Aegean region continues to struggle with a dynamic climate marked by recurring droughts and crop failures [68,110].

#### 5.2.4 Moderate Climatic State (240–260 CE, Snapshot 4).

The fourth snapshot depicts a moderate climatic state characterized by above-average temperatures and slightly above-average precipitation forcing. This scenario represents the highest extent of agricultural suitability observed in the model, indicated by warm and somewhat wet conditions that are conducive to the cultivation of diverse crops across both central and peripheral areas. However, due to the difference between the MPI-ESM simulation and the pollen-based climate reconstruction for the fourth snapshot, the dynamics of our model may not fully capture historical developments. Nevertheless, if such warmer conditions, as indicated in the climate model and reconstructions, coincided with a period of urban stagnation in Pergamon [42], they may have supported sustained agricultural productivity in rural areas, even as the urban population declined.

The third snapshot (100–120 CE), despite being characterized by warmer and drier conditions, exhibited an overall lower suitability compared to the fourth snapshot. Similarly, the first snapshot (310–290 BCE), which was colder and wetter, also showed reduced suitability. These patterns suggest that neither increased rainfall nor elevated temperatures alone were sufficient to maximize suitability. Instead, the combination of moderately increased precipitation and warmer temperatures, as seen in the fourth snapshot, appears to have provided conditions supporting broader agricultural expansion, including in areas previously less suitable for cultivation. Favorable climatic conditions could have enhanced crop yields and expanded the range of cultivable areas, potentially mitigating some economic challenges associated with urban stagnation. This scenario suggests that agricultural cultivation may have remained robust or thrived independently of urban dynamics, highlighting a potential decoupling between urban development and rural agricultural productivity during this period [20,111].

Here, the divergence between the MPI-ESM simulation and the pollen-based reconstruction highlights the complexity of reconstructing past climatic conditions, particularly in regions with diverse microclimates. While both datasets indicate warmer conditions, differences in precipitation patterns underscore the need to interpret the model outputs carefully. To contextualize the modeled scenarios, this study addresses these uncertainties by integrating additional evidence, including comparisons with the Elaia pollen record and archaeological surveys. While these external data sources provide complementary perspectives, they also highlight the inherent challenges of reconstructing historical socio-ecological systems based on incomplete records [112,113].

The sustained agricultural suitability indicated by the fourth snapshot aligns with the possibility of continued or intensified rural agricultural activities during urban decline. If these conditions prevailed, rural communities may have capitalized on the favorable climate to maintain local economies and potentially engage in regional trade networks, contributing to their resilience despite challenges faced by urban centers like Pergamon [114].

#### 5.2.5 Late Roman Period (340–360 CE, Snapshot 5).

The fifth snapshot reflects a slightly warmer-than-average but drier climatic state, with variability in precipitation. While overall agricultural suitability remained moderate, the combination of lower temperatures and reduced precipitation compared to the previous snapshot constrained cultivation and limited the potential for agricultural expansion. The modeled results indicate that core agricultural areas, such as the western Bakırçay plain, continued to show moderate suitability for cultivation. In contrast, marginal areas experienced a reduction in suitability compared to earlier snapshots. Notably, the trends indicated by the MPI-ESM simulation align with the broader patterns suggested by the pollen-based reconstruction for this period. Both datasets reflect drier conditions, reinforcing the plausibility of the climate simulated in this snapshot. Despite these challenges, some regions with favorable topographic and edaphic conditions may have retained their suitability for less water-dependent crops. Despite these climatic challenges, evidence for settlement expansion during the 4th century CE suggests a more complex interaction between environmental conditions and socio-economic dynamics [115]. For this scenario, the renewed expansion of settlements during this period may have been influenced by shifts in regional trade networks and the broader economic landscape of western Anatolia. Drier conditions may have necessitated an increased reliance on trade to supplement local agricultural production, particularly in regions where crop yields declined. The enhanced trade connectivity during Roman rule [108] may have enabled settlements within the Pergamon micro-region to access resources beyond their immediate vicinity, thereby mitigating the impacts of climatic constraints and supporting population growth or redistribution.

### 5.3 Adaption and Risk Management

The higher modeled agricultural suitability of the western plain compared to the more humid eastern plain raises the question of whether increased precipitation, typically considered beneficial for agriculture within a Mediterranean Csa climate, enhances agricultural potential.

In climates classified as Csa, where dry summers and wet winters typify the agricultural cycle, the interaction between precipitation and temperature plays a critical role in determining agricultural suitability [68]. While precipitation is generally essential for crop growth, its effectiveness depends on timing and intensity. In this context, the modeled results suggest that moderate increases in average annual precipitation, when combined with higher average annual temperatures, may create optimal conditions for agriculture in the environs of Pergamon. These conditions likely reduce water stress during the growing season while simultaneously extending the range of suitable areas for cultivation.

During the wet winter months (typically October to May), the cooler temperatures in a Mediterranean Csa climate minimize water loss through evaporation, making precipitation during this period particularly effective for replenishing soil moisture. The high frequent winter rains [116] often promote, rather than constrain, agricultural productivity by providing much-needed moisture during the typical growing period for annual crops [68]. However, as observed in the more humid eastern plain, excessive humidity or precipitation may reduce suitability by increasing the risk of waterlogging and limiting the cultivable area, particularly for crops sensitive to overly wet conditions.

The results of the agricultural model highlight the importance of balanced precipitation and temperature conditions. A suitable combination of these factors, as seen in the western plain, appears to enhance agricultural potential by reducing the dependency on extreme climatic conditions. However, the Mediterranean climate’s reliance on the rainy season remains a challenge for long-term agricultural planning, as seasonal shifts or interruptions in precipitation patterns can have severe impacts on crop yields and sustainability [117].

It is nevertheless important to consider that higher annual temperatures, particularly in the western plain, may have a more pronounced impact on the cultivation suitability at the regional level. On the one hand, warmer conditions can extend growing seasons in higher elevations, enabling the cultivation of a wider variety of crops and even increasing yields for certain heat-tolerant species. On the other hand, higher temperatures can also lead to faster phenological development and, therefore, shortening of growing periods [118]. The benefits of longer growing periods must also be weighed against the risks of prolonged dry spells during the summer months, which could stress crops if water resources are not carefully managed and utilized.

Furthermore, winter precipitation in the Aegean region often occurs as extreme events [119], leading to surface runoff, soil erosion, and damage to agricultural fields. Only a small fraction infiltrates in the soil and becomes available for plants [120]. Land degradation poses a challenge to land management and long-term soil fertility [121]. While these extreme events can have a severe impact on the landscape and agricultural practices, their effects are difficult to capture in climate analyses that rely on monthly averages, which tend to smooth out these extremes.

Accordingly, while temperature and precipitation both substantially influence the region’s suitability for agriculture, in this particular Mediterranean context, higher annual temperatures appear to have a more positive impact on the region’s agricultural systems. However, the unpredictability and erosive impact of extreme precipitation events remains a critical challenge, particularly in areas prone to soil degradation and runoff [121].

While the favorable cultivation conditions – and, therefore, a presumable higher species equitability – offered advantages for agriculture in the western lower Bakırçay plain, the exploitation of these favorable conditions likely increased pressure on the landscape, particularly on the adjacent slopes and the plain soils [12,51,66]. Intensive agricultural practices, typical of antiquity [76], may have contributed to soil degradation and erosion, creating negative feedback loops that increased the landscape’s sensitivity to environmental changes [10,11,66]. This degradation would have been particularly pronounced in areas associated with monocropping and limited crop rotation – practices attributed to some Roman agriculture systems, especially in zones of intensified production [51,76]. However, these practices cannot be generalized across the Roman Empire. The diversity of land tenure systems, production scales, and ecological settings across the Roman Empire suggests a spectrum of cultivation strategies – including mixed cropping and fallow-based systems – that complicates simplistic assumptions [15,104,122]. In the context of urban centers like Pergamon, agricultural organization may have been shaped as much by demand and logistical networks as by environmental conditions.

While such socio-economic diversity complicates attempts to generalize cultivation strategies, it also underscores the value of analytically separating ecological potential from actual land use. Therefore, the presented models do not attempt to reconstruct agricultural systems or specific cropping decisions but instead focus on the environmental suitability for cultivation under different climatic scenarios. This distinction is particularly relevant when contrasting the relatively stable conditions of the lower plain with the more heterogeneous landscapes of the surrounding mountain ranges.

Mountainous areas, located at a distance from the coastal zones in the eastern part of the Pergamon micro-region, present a contrasting scenario to the high suitability in the western, lower Bakırçay plain. These regions, characterized by frequent changes in cultivation suitability (Fig 7), would have required more adaptive strategies to cultivate crops and maintain food security. Crops like wheat, barley, and lentils appear more resilient to these fluctuations, allowing for continued cultivation despite climatic shifts (Figs 5 and 6). In contrast, other crops, such as emmer, durum wheat, and bitter vetch, showed high variability in their suitability over time (Fig 7), particularly in the foothills and intermountain basins surrounding the plain (Fig 5). The variability of these crops, especially durum wheat, was most pronounced in the foothills northeast of Elaia and Pergamon, where agroclimatic conditions fluctuated markedly. Therefore, for the mountainous parts of the Pergamon micro-region, adaptive strategies such as intercropping may have played a crucial role in creating a more resilient system and reducing the risk of crop failures [15,68]. The extent to which trade influenced food security and the prosperity of the Pergamon micro-region is unknown. This shifts the focus from local water availability to broader networked dimensions, including concepts like virtual water [123]. Here, not only would actual agricultural products be considered for trade across the nodes of superregional networks, but also regional input parameters, such as soil nutrients, available water, and labor, that facilitate the growth and enhance the productivity of a specific crop.

Historical examples of adaptive processes related to climatic fluctuations are found in the south and west of Smyrna (approximately 100 km south of Pergamon). Here, the archaeobotanical study of Maltas et al. (2023) indicates a decline in the cultivation of glume wheat (einkorn and emmer) and a notable increase in barley between the Early Bronze Age (ca. 3100/3000–2600 BCE) and the Middle Bronze Age (~2140–1890 BCE) [2]. The authors suggest that this shift reflects an adaptive strategy in response to climatic variability, as barley is a drought-resistant cereal alternative to glume wheat. Compared to our modeling results – which cover a time frame approximately 1500 years later – barley is identified as the most suitable and stable crop among all the modeled taxa (Figs 5 and 6). Although our models indicate that emmer had limited suitability for cultivation in this region, the comparison between the various climatic forcings demonstrates only a marginal influence on the natural potential of emmer, suggesting a rather resilient natural suitability. It should be noted, however, that our models do not account for soil enrichment and other forms of melioration, which may contribute to the declining resistance of such marginal crops in actual practice. In addition, the records around Smyrna indicate that legumes, such as bitter vetch, were cultivated in greater quantities, while the amount of lentils decreased over the periods [2]. Although areas occupy a large portion of moderately to highly suitable areas in our study (Fig 5) barley shows fewer fluctuations in suitability across different climatic forcing scenarios per snapshot. Lentil and bitter vetch demonstrate a notable shift in suitability, particularly in the second snapshot (10 BCE – 10 CE).

## 6 Conclusions

In this study, we examined the agroclimatic potential in the environs of the ancient city of Pergamon, focusing on the spatio-temporal variability in suitability for cultivating various crops and its relationship to the historical transformation of the Pergamon micro-region. The different modeled climatic settings had implications for the agricultural potential within the micro-region with a clear spatial differentiation. Comparing the forcing based MPI-ESM climate simulation to a pollen-based reconstruction of climatic variables revealed general climatic alignments between the two methods. Our modeling approach identifies different climatic settings that supported agricultural potential. Thus, we could differentiate potential socio-ecological dynamics that might have accompanied the development of Pergamon from a Hellenistic polis to a Roman metropolis. Here, dynamics such as integration into the wider Roman trade network might have decoupled Pergamon’s Hellenistic subsistence farming system from environmental reliance. At the same time, newly adapted dependencies on external supplies could have introduced vulnerabilities to disruptions in trade. The comparison of the local cereal pollen record from Elaia emphasizes that agricultural expansion and suitability are not directly linked despite the limited comparability of pollen records and climate simulations due to ecological feedback.

Our heuristic approach does thus not fully account for the overall complexity of the changing social metabolism during Pergamon’s transformation from a Hellenistic polis to a Roman metropolis. In conclusion, it can be assumed that the perception of changing cultivation suitability for various crops must have led to the implementation of different land management systems across the Pergamon micro-region. Our findings indicate that the western Bakırçay plain was particularly important for consistent crop production, largely due to favorable climatic influences that ensured stable conditions for various crops. However, this stability was accompanied by challenges, such as accelerated soil degradation, particularly in the plains and adjacent slopes, which required management strategies to maintain long-term agricultural productivity. This may be especially true in areas with consistently high suitability, particularly during periods of high population pressure and declining suitability for cultivation. In contrast, the more variable cultivation suitability in the eastern plain, as well as in the mountain ranges and foothills of the Pergamon micro-region, demanded more adaptive strategies to navigate climatic fluctuations across a lower variety of crops. This emphasizes the role of localized agroclimatic conditions and adaptive land management in shaping ancient strategies for food security under shifting Mediterranean climates. Furthermore, our findings question the notion of a “Roman” Climate Optimum, suggesting that regional climatic fluctuations were a prominent factor in determining suitable agricultural practices across different landscape units. In the case of the Pergamon micro-region, this variability directly influenced agricultural potential and likely shaped adaptive responses. These insights emphasize the need to move beyond climatic narratives and instead adopt differentiated, scale-sensitive perspectives when evaluating socio-ecological systems. As for today, western Anatolia remains a blank spot for isotopic climate proxies such as δ¹⁸O records. Future research could benefit from incorporating higher-resolution paleoclimatic data derived from proxies such as lake sediment cores, speleothems, or additional pollen sequences, as well as more detailed archaeological datasets, including archaeobotanical and settlement evidence, to refine and enhance such models. Furthermore, an expansion of the study’s spatial and temporal scope could provide deeper insights into the long-term agroclimatic trends that shaped the western Anatolian coastline.

## Supporting information

S1 File R-script for model implementation. Code used to apply the model and define parameter settings.
(HTML)
