# Supplementary material for: Agroclimatic landscapes of pergamon: Modeling agricultural suitability of an ancient city and its environs
Source: PLoS One. 2025 Jul 2;20(7):e0325779. doi: 10.1371/journal.pone.0325779 (PMC12221027; doi:10.1371/journal.pone.0325779)
Supplement: S1 File — Code used to apply the model and define parameter settings. (HTML) [file pone.0325779.s001.html]

Supplementary Material - Agroclimatic Landscapes of Pergamon


# Supplementary Material - Agroclimatic Landscapes of Pergamon

#### Robert Busch

#### 2025-04-14

Abstract

This script accompanies the research study, “Agroclimatic Landscapes
of Pergamon: Modeling Agricultural Suitability of an Ancient City and
its Environs,” as supplementary material. It includes the methodology
for calculating and classifying the ecological suitability of various
cereal and legume species based on soil, climate, and topographic data.
The datasets used in this study are available at DOI:
10.5281/zenodo.14551863

- 1 Set up the project
- 2 Read processed data
  - 2.1 Topography
    - 2.1.1 Digital Elevation Model
    - 2.1.2 Slope
    - 2.1.3 Sun exposure
    - 2.1.4 Wind exposure
    - 2.1.5 Topographic Wetness
      Index
  - 2.2 Soil
    - 2.2.1 Soil texture
    - 2.2.2 Soil pH
  - 2.3 Climate
    - 2.3.1 Downscaled temperature
    - 2.3.2 Downscaled
      precipitation
- 3 EcoCrop set up
  - 3.1 Soil fuzzy ranges
  - 3.2 EcoCrop Models
  - 3.3 EcoCrop classifier
- 4 Eco Crop models
  - 4.1 Cereals
    - 4.1.1 Common wheat - Triticum
      aestivum
    - 4.1.2 Durum wheat - Triticum
      Durum
    - 4.1.3 Barley - Hordeum
      vulgare
    - 4.1.4 Emmer - Triticum
      dicoccon
  - 4.2 Legumes
    - 4.2.1 Lentil - Lens culinaris
      medikus
    - 4.2.2 Bitter vetch - Vicia
      ervilia
  - 4.3 Hybrid model

# 1 Set up the project

Install and update packages

```
# Display R version information (for this script we used R version 4.3.3)
R.version.string

# List of required packages
packages <- c("dplyr", "knitr","raster", "sf","ncdf4", "tidyr","pals","rasterVis","Recocrop", "rcartocolor", "scico")

# Install missing packages
packages_new <- packages[!(packages %in% installed.packages()[, "Package"])]
if (length(packages_new) > 0) {
  install.packages(packages_new, repos = "http://cran.us.r-project.org")
}

# Load packages
lapply(packages, require, character.only = TRUE)

# Clear all objects from the workspace and free up memory
rm(list = ls(all.names = TRUE))
gc()
```

# 2 Read processed data

## 2.1 Topography

All data was resampled to the same resolution as of WorldClim 2.1
data during preprocessing in ArcGIS. Nearest neighbor resampling was
used.

### 2.1.1 Digital Elevation Model

**Digital Elevation Model** based on TanDEM-X data. The
data was cropped using ArcGIS.

```
DEM             <- as(raster(DEM_dir),"SpatRaster")        
levelplot(DEM,col.regions = rev(carto_pal(20, "Earth")),
          margin =FALSE,main="Digital Elevation Model")
```

[m above sea level]

### 2.1.2 Slope

**Slope** derivative of the TanDEM-X data, calculated in
ArcGIS
Pro.

```
SLOPE           <- as(raster(SLOPE_dir),"SpatRaster")    
levelplot(SLOPE,col.regions = rev(carto_pal(20, "Sunset")),
          margin =FALSE,main="Slope")
```

[°]

### 2.1.3 Sun exposure

**Sun exposure** derivative of the TanDEM-X data,
calculated in ArcGIS
Pro.

```
RADI            <- as(stack(RADI_dir),"SpatRaster")    
month           <- c("Jan","Feb","Mar","Apr","May","Jun","Jul","Aug","Sep","Oct","Nov","Dec")
names(RADI)     <- month
levelplot(RADI,col.regions = rev(carto_pal(20, "Sunset")),
          margin =FALSE,main="Sun exposure")
```

[h/d]

### 2.1.4 Wind exposure

**Wind exposure** derivative of the TanDEM-X data,
calculated in SAGA GIS as Wind
Exposition Index.

```
WIND            <- as(raster(WIND_dir),"SpatRaster")     
levelplot(WIND,col.regions = carto_pal(20, "Teal"),
          margin =FALSE,main="Wind Exposure")
```

### 2.1.5 Topographic Wetness Index

**Topographic Wetness Index (TWI)** derivative of the
TanDEM-X data, calculated in SAGA GIS as SAGA
Wetness Index.

```
TWI             <- as(raster(TWI_dir),"SpatRaster")      
levelplot(TWI,col.regions = carto_pal(20, "Earth"),
          margin =FALSE,main="Topographic Wetness")
```

## 2.2 Soil

**Soil data** from the SoilGrids (R function: geodata::soil\_world).

### 2.2.1 Soil texture

```
SOIL            <- as(stack(SOIL_dir),"SpatRaster")
names(SOIL)     <- c("sand","silt","clay","pH")
levelplot(SOIL[[1:3]],col.regions = rev(carto_pal(20, "Sunset")),
          margin =FALSE,main="Soil Texture")
```

[vol. %]

### 2.2.2 Soil pH

```
levelplot(SOIL[[4]],col.regions = rev(carto_pal(20, "Sunset")),
          margin =FALSE,main="Soil pH")
```

## 2.3 Climate

**Climate data** downscaled MPI-ESM 1.2 climate data.
The MPI-ESM 1.2 data was statistically downscaled using the
delta-method. We used the WorldClim 2.1
30’’ to downscale the data. The processing was computed in the High Performance Computer
System for Earth System Research (HLRE-4) “Levante” availabe by the
Deutsche Klimarechenzentrum (DKRZ). The code for this process is not
included.

### 2.3.1 Downscaled temperature

```
MPI_ESM_temp        <- as(stack(MPI_ESM_temp_dir),"SpatRaster")

#First snapshot as a data example. 
MPI_ESM_temp_subset <- MPI_ESM_temp[[1:12]]
names(MPI_ESM_temp_subset) <- month
levelplot(MPI_ESM_temp_subset,col.regions = carto_pal(20, "Geyser"),
          main="Downscaled Temperature (310-290 BCE)")
```

[°C/month]

### 2.3.2 Downscaled precipitation

```
MPI_ESM_precip  <- as(stack(MPI_ESM_precip_dir),"SpatRaster") 

#First snapshot as a data example.
MPI_ESM_precip_subset <- MPI_ESM_precip[[1:12]]
names(MPI_ESM_precip_subset) <- month
levelplot(MPI_ESM_precip_subset,col.regions = rev(carto_pal(20, "Geyser")),
          main="Downscaled Precipitation (310-290 BCE)")
```

[mm/month]

# 3 EcoCrop set up

## 3.1 Soil fuzzy ranges

We created fuzzy ranges for different soil types. The EcoCrop model
only differentiate between light and heavy soils. Therefore we had to
parameterize these soils into soil texture compositions [vol. %]. This
represents our own estimation. Optimal and Absolute ranges of soil
textures vary between different crops. Soil textures are indicated as
c(abosulte minimum,optimal minimum,optimal maximum,absolute
maximum).

```
soil_medium_heavy <- cbind(clay=c(20,20,30,40),silt=c(20,30,40,50),sand=c(20,30,40,50))

soil_medium <- cbind(clay=c(10,20,30,40),silt=c(20,30,40,50),sand=c(20,30,40,50))

soil_medium_light_to_heavy <- cbind(clay=c(10,10,20,40),silt=c(20,30,40,50),sand=c(20,30,50,60))

soil_medium_light_light_to_heavy <- cbind(clay=c(5,10,20,40),silt=c(10,20,30,40),sand=c(20,40,50,60))

soil_medium_heavy_light_to_heavy <- cbind(clay=c(30,40,50,70),silt=c(20,30,40,50),sand=c(10,20,30,40))

soil.list <- list(soil_medium_heavy,
                  soil_medium,
                  soil_medium_light_to_heavy,
                  soil_medium_light_light_to_heavy,
                  soil_medium_heavy_light_to_heavy)

names(soil.list) <- c("soil_medium_heavy",
                  "soil_medium",
                  "soil_medium_light_to_heavy",
                  "soil_medium_light_light_to_heavy",
                  "soil_medium_heavy_light_to_heavy")
```

## 3.2 EcoCrop Models

Setting up a list containing all EcoCrop model using the Recocrop
package.

```
fname <- system.file("parameters/ecocrop.rds", package="Recocrop")

# list with all ecocrop taxa in the Recocrop package
d <- readRDS(fname) 

# list of all crops
crops <- c("Wheat, common",
           "emmer",
           "Triticum durum",
           "Barley",
           "Lens culinaris",
           "Vicia ervilia"
           )

fuzzy_crops <- list()

for (a in 1:length(crops)) {
  
  crop_temp <- Recocrop::ecocropPars(crops[a]) 

  crop_p <- crop_temp$parameters

  eco_mod <- ecocrop(crop_p)
  
  crop(eco_mod) <- crop_p
  
# Static slope ranges adapted after Laabs & Knitter (2021)  
  crop(eco_mod) <- cbind(slope = c(0,0,8,15)) 
  
# Static wind ranges based on current land use.Values below 1 indicate wind-shielded areas; values above 1, wind-exposed areas
  crop(eco_mod) <- cbind(wind=c(0,0,1,1.15))  
  
# Static TWI ranges adapted after Laabs & Knitter (2021)
  crop(eco_mod) <- cbind(wetness=c(0,6,7,16)) 
  
  fuzzy_crops[[a]] <- eco_mod
}

names(fuzzy_crops) <- crops

#set up for loops and maps
snapshots <- list(c(1:12),#310 - 290 BCE 
                  c(13:24),#10 BCE - 10 CE
                  c(25:36),#100 - 120 CE
                  c(37:48),#240 - 260 CE
                  c(49:60))#340 - 360 CE

timeframes <- c("310-290BCE", "10BCE-10CE","100-120CE", "240-260CE", "340-360CE")

col <- ocean.tempo(100)

rm(a,d,crop_temp,fname,eco_mod,crop_p)
```

## 3.3 EcoCrop classifier

Creating an classifier that classifies estimated EcoCrop potentials
into classes from very low to very high suitable

```
# Median Absolute Deviation (MAD) 
mad_func <- function(x) {
  median(abs(x - median(x, na.rm=TRUE)), na.rm=TRUE)
  }

eco_classifier<- function(x){

  x_scale <- x/max(maxValue(x)) # scale dataset individually
    
  # Reclassify each layer in the raster stack based on the defined thresholds
  mat <- c(0,0,0,                     # NA
           0.00000000000000001,0.2,1, # very low
           0.200000000000000001,0.4,2,# low
           0.400000000000000001,0.6,3,# moderate
           0.600000000000000001,0.8,4,# high
           0.800000000000000001,1,5)  # very high
  
  rclmat <- matrix(mat, ncol=3, byrow=TRUE) 
  
  reclassified_stack <- stack() 
  
  # Apply Reclassification
  for (i in 1:nlayers(x)) { 
    layer <- x_scale[[i]]
    reclassified_layer <- reclassify(layer, rclmat,right=FALSE)
    reclassified_stack <- addLayer(reclassified_stack, reclassified_layer)
  }
  
  # median-centered coefficient of variation
  mad_cv <- calc(x_scale, function(y) (mad_func(y) / median(y, na.rm=TRUE)) * 100) 
  
  # mean-centered coefficient of variation
  mean_cov <- (calc(x_scale,sd)/calc(x_scale,mean))*100                            
  
  reclassified_stack <- addLayer(reclassified_stack, mad_cv)
  
  reclassified_stack <- addLayer(reclassified_stack, mean_cov)

  names(reclassified_stack) <- c(timeframes, "COV_median[%]","COV_mean[%]")
  
  return(reclassified_stack)
}
```

# 4 Eco Crop models

## 4.1 Cereals

### 4.1.1 Common wheat - Triticum aestivum

```
# Crop-specific parameters

# Soil parameters
crop(fuzzy_crops$`Wheat, common`) <- soil.list$soil_medium_heavy 

# Photoperiod
crop(fuzzy_crops$`Wheat, common`) <- cbind(photop=c(1,12,24,24))

# set growing time according to EcoCrop
fuzzy_crops$`Wheat, common`$duration <- 90 

#create empty lists, which will be filled with the models of the individual time steps
wheat_stack_mean <- stack() 

#set up the model
control(fuzzy_crops$`Wheat, common`, get_max=TRUE) 

for (xy in c(1:5)) { x <- predict(fuzzy_crops$`Wheat, common`,
                            tavg = MPI_ESM_temp[[snapshots[[xy]]]],#dynamic
                            prec = MPI_ESM_precip[[snapshots[[xy]]]],#dynamic
                            wetness = TWI[[1]], #static
                            slope = SLOPE[[1]], #static
                            clay = SOIL[[3]],#static
                            silt = SOIL[[2]],#static
                            sand = SOIL[[1]],#static
                            ph = SOIL[[4]],#static
                            photop = RADI,#semi-static
                            wind = WIND[[1]])#static
# transform to raster
x_rast <- raster(x) 
#Add new snapshot to the stack                    
wheat_stack_mean <- addLayer(wheat_stack_mean,x_rast)           

# Name according to the snapshot
names(wheat_stack_mean[[xy]]) <- timeframes[xy] 
 }
# Classify the EcoCrop estimations
wheat_stack_mean_classified <- eco_classifier(wheat_stack_mean)

levelplot(wheat_stack_mean_classified[[1:5]],col.regions = col)
```

```
# Plot tolerance ranges for crop-specific environmental parameters
#(average temperature, precipitation, pH, Slope, Wind Exposure, Topographic wetness, Clay content, Silt content, Sand content, Solar radiation)
plot(fuzzy_crops$`Wheat, common`)
```

### 4.1.2 Durum wheat - Triticum Durum

```
crop(fuzzy_crops$`Triticum durum`) <- soil.list$soil_medium_heavy 

crop(fuzzy_crops$`Triticum durum`) <- cbind(photop=c(1,12,24,24)) 

fuzzy_crops$`Triticum durum`$duration <- 120 

durum_stack_mean <- stack()

control(fuzzy_crops$`Triticum durum`, get_max=TRUE) 

for (xy in c(1:5)) {   x <- predict(fuzzy_crops$`Triticum durum`,
                            tavg = MPI_ESM_temp[[snapshots[[xy]]]],#dynamic
                            prec = MPI_ESM_precip[[snapshots[[xy]]]],#dynamic
                            wetness = TWI[[1]],#static
                            slope = SLOPE[[1]],#static
                            clay = SOIL[[3]],#static
                            silt = SOIL[[2]],#static
                            sand = SOIL[[1]],#static
                            ph = SOIL[[4]],#static
                            photop = RADI,#semi-static
                            wind = WIND[[1]])#static

x_rast <- raster(x) 
                   
durum_stack_mean <- addLayer(durum_stack_mean,x_rast)           
  
names(durum_stack_mean[[xy]]) <- timeframes[xy] 
}

drurum_stack_mean_classified <- eco_classifier(durum_stack_mean)

levelplot(drurum_stack_mean_classified[[1:5]],col.regions = col)
```

```
# Plot tolerance ranges for crop-specific environmental parameters
#(average temperature, precipitation, pH, Slope, Wind Exposure, Topographic wetness, Clay content, Silt content, Sand content, Solar radiation)
plot(fuzzy_crops$`Triticum durum`)
```

### 4.1.3 Barley - Hordeum vulgare

```
crop(fuzzy_crops$Barley) <- soil.list$soil_medium_light_to_heavy

crop(fuzzy_crops$Barley) <- cbind(photop=c(1,12,24,24))

fuzzy_crops$Barley$duration <- 90

control(fuzzy_crops$Barley, get_max=TRUE)

hordeum_stack_mean <- stack()

for (xy in c(1:5)) {   x <- predict(fuzzy_crops$Barley,
                            tavg = MPI_ESM_temp[[snapshots[[xy]]]],#dynamic
                            prec = MPI_ESM_precip[[snapshots[[xy]]]],#dynamic
                            wetness = TWI[[1]],
                            slope = SLOPE[[1]],#static
                            clay = SOIL[[3]],#static
                            silt = SOIL[[2]],#static
                            sand = SOIL[[1]],#static
                            ph = SOIL[[4]],#static
                            photop = RADI,#semi-static
                            wind = WIND[[1]])#static

x_rast <- raster(x) 
                   
hordeum_stack_mean <- addLayer(hordeum_stack_mean,x_rast)           
  
names(hordeum_stack_mean[[xy]]) <- timeframes[xy]
}

hordeum_stack_mean_classified <- eco_classifier(hordeum_stack_mean)

levelplot(hordeum_stack_mean_classified[[1:5]],col.regions = col)
```

```
# Plot tolerance ranges for crop-specific environmental parameters
#(average temperature, precipitation, pH, Slope, Wind Exposure, Topographic wetness, Clay content, Silt content, Sand content, Solar radiation)
plot(fuzzy_crops$Barley)
```

### 4.1.4 Emmer - Triticum dicoccon

```
crop(fuzzy_crops$emmer) <- soil.list$soil_medium_light_light_to_heavy

crop(fuzzy_crops$emmer) <- cbind(photop=c(1,12,24,24))

fuzzy_crops$emmer$duration <- 120

control(fuzzy_crops$emmer, get_max=TRUE)

dicoccon_stack_mean <- stack()

for (xy in c(1:5)) {   x <- predict(fuzzy_crops$emmer,
                            tavg = MPI_ESM_temp[[snapshots[[xy]]]],#dynamic
                            prec = MPI_ESM_precip[[snapshots[[xy]]]],#dynamic
                            wetness = TWI[[1]], #static
                            slope = SLOPE[[1]],#static
                            clay = SOIL[[3]],#static
                            silt = SOIL[[2]],#static
                            sand = SOIL[[1]],#static
                            ph = SOIL[[4]],#static
                            photop = RADI,#semi-static
                            wind = WIND[[1]])#static

x_rast <- raster(x) 
                   
dicoccon_stack_mean <- addLayer(dicoccon_stack_mean,x_rast)           
  
names(dicoccon_stack_mean[[xy]]) <- timeframes[xy] 
}

dicoccon_stack_mean_classified <- eco_classifier(dicoccon_stack_mean)

levelplot(dicoccon_stack_mean_classified[[1:5]],col.regions = col)
```

```
# Plot tolerance ranges for crop-specific environmental parameters
#(average temperature, precipitation, pH, Slope, Wind Exposure, Topographic wetness, Clay content, Silt content, Sand content, Solar radiation)
plot(fuzzy_crops$emmer)
```

## 4.2 Legumes

### 4.2.1 Lentil - Lens culinaris medikus

```
crop(fuzzy_crops$`Lens culinaris`) <- soil.list$soil_medium_heavy_light_to_heavy

crop(fuzzy_crops$`Lens culinaris`) <- cbind(photop=c(1,12,24,24))

fuzzy_crops$`Lens culinaris`$duration <- 70

control(fuzzy_crops$`Lens culinaris`, get_max=TRUE)

lens_stack_mean <- stack()

for (xy in c(1:5)) {   x <- predict(fuzzy_crops$`Lens culinaris`,
                            tavg = MPI_ESM_temp[[snapshots[[xy]]]],#dynamic
                            prec = MPI_ESM_precip[[snapshots[[xy]]]],#dynamic
                            wetness = TWI[[1]], #static
                            slope = SLOPE[[1]],#Static
                            clay = SOIL[[3]],#static
                            silt = SOIL[[2]],#static
                            sand = SOIL[[1]],#static
                            ph = SOIL[[4]],#static
                            photop = RADI,#semi-static
                            wind = WIND[[1]])#static

x_rast <- raster(x)
                   
lens_stack_mean <- addLayer(lens_stack_mean,x_rast)           
  
names(lens_stack_mean[[xy]]) <- timeframes[xy] 
}

lens_stack_mean_classified <- eco_classifier(lens_stack_mean)

levelplot(lens_stack_mean_classified[[1:5]],col.regions = col)
```

```
# Plot tolerance ranges for crop-specific environmental parameters
#(average temperature, precipitation, pH, Slope, Wind Exposure, Topographic wetness, Clay content, Silt content, Sand content, Solar radiation)
plot(fuzzy_crops$`Lens culinaris`)
```

### 4.2.2 Bitter vetch - Vicia ervilia

```
crop(fuzzy_crops$`Vicia ervilia`) <-soil.list$soil_medium_light_light_to_heavy

crop(fuzzy_crops$`Vicia ervilia`) <- cbind(photop=c(1,12,24,24))

fuzzy_crops$`Vicia ervilia`$duration <- 90

control(fuzzy_crops$`Vicia ervilia`, get_max=TRUE)

ervilia_stack_mean <- stack()

for (xy in c(1:5)) {   x <- predict(fuzzy_crops$`Vicia ervilia`,
                            tavg = MPI_ESM_temp[[snapshots[[xy]]]],#dynamic
                            prec = MPI_ESM_precip[[snapshots[[xy]]]],#dynamic
                            wetness = TWI[[1]], #static
                            slope = SLOPE[[1]],
                            clay = SOIL[[3]],#static
                            silt = SOIL[[2]],#static
                            sand = SOIL[[1]],#static
                            ph = SOIL[[4]],#static
                            photop = RADI,#semi-static
                            wind = WIND[[1]])#static

x_rast <- raster(x) 
                   
ervilia_stack_mean <- addLayer(ervilia_stack_mean,x_rast)         
  
names(ervilia_stack_mean[[xy]]) <- timeframes[xy]
}

ervilia_stack_mean_classified <- eco_classifier(ervilia_stack_mean)

levelplot(ervilia_stack_mean_classified[[1:5]],col.regions = col)
```

```
# Plot tolerance ranges for crop-specific environmental parameters
#(average temperature, precipitation, pH, Slope, Wind Exposure, Topographic wetness, Clay content, Silt content, Sand content, Solar radiation)
plot(fuzzy_crops$`Vicia ervilia`)
```

## 4.3 Hybrid model

The **hybrid model** is aimed to estimate areas that
were suitable for the combination of several crops and therefore made
crop cycles, intercropping and further measures possible.

```
crops.list <- list(wheat_stack_mean,
     durum_stack_mean,
     hordeum_stack_mean,
     dicoccon_stack_mean,
     lens_stack_mean,
     ervilia_stack_mean
     )

names(crops.list) <- c("Wheat, common",
                       "Triticum durum",
                       "Barley",
                       "emmer",
                       "Lens culinaris",
                       "Vicia ervilia"
                       )

hybrid <- stack()

# Classify areas across each snapshots which were at least "low" suitable for cultivation among the seven crops below
for (x in c(1:5)) {
raster_temp <- (crops.list$`Wheat, common`[[x]]/max(maxValue(crops.list$`Wheat, common`)))>0.2 &
               (crops.list$`Triticum durum`[[x]]/max(maxValue(crops.list$`Triticum durum`)))>0.2  &
               (crops.list$Barley[[x]]/max(maxValue(crops.list$Barley)))>0.2 &
               (crops.list$emmer[[x]]/max(maxValue(crops.list$emmer)))>0.2 &
               (crops.list$`Vicia ervilia`[[x]]/max(maxValue(crops.list$`Vicia ervilia`)))>0.2 &
               (crops.list$`Lens culinaris`[[x]]/max(maxValue(crops.list$`Lens culinaris`)))>0.2

hybrid <- addLayer(hybrid,raster_temp)

}

names(hybrid) <- timeframes

levelplot(hybrid[[1:5]],col.regions = c("#FFFFFF",col[50]),colorkey = FALSE) #green=suitable/white=unsuitable
```
